# Review on Nanoparticles and Nanostructured Materials: Bioimaging, Biosensing, Drug Delivery, Tissue Engineering, Antimicrobial, and Agro-Food Applications

**DOI:** 10.3390/nano12030457

**Published:** 2022-01-28

**Authors:** Vancha Harish, Devesh Tewari, Manish Gaur, Awadh Bihari Yadav, Shiv Swaroop, Mikhael Bechelany, Ahmed Barhoum

**Affiliations:** 1 School of Pharmaceutical Sciences, Lovely Professional University, Phagwara, Punjab 144401, India; vanchaharish@gmail.com (V.H.); dtewari3@gmail.com (D.T.); 2 Centre of Biotechnology, University of Allahabad, Prayagraj, Uttar Pradesh 211002, India; manishgaur@allduniv.ac.in; 3 Department of Biochemistry, Central University of Rajasthan, Ajmer 305817, India; shivswaroop@curaj.ac.in; 4 Institut Européen des Membranes, IEM UMR 5635, University Montpellier, ENSCM, CNRS, 34730 Montpellier, France; 5 NanoStruc Research Group, Chemistry Department, Faculty of Science, Ain Helwan, Cairo 11795, Egypt; 6 National Centre for Sensor Research, School of Chemical Sciences, Dublin City University, D09 Y074 Dublin, Ireland

**Keywords:** nanostructures, nanomaterials, drug delivery systems, tissue-engineered scaffolds, wound dressings, skincare, risks and toxicities, market and regulations

## Abstract

In the last few decades, the vast potential of nanomaterials for biomedical and healthcare applications has been extensively investigated. Several case studies demonstrated that nanomaterials can offer solutions to the current challenges of raw materials in the biomedical and healthcare fields. This review describes the different nanoparticles and nanostructured material synthesis approaches and presents some emerging biomedical, healthcare, and agro-food applications. This review focuses on various nanomaterial types (e.g., spherical, nanorods, nanotubes, nanosheets, nanofibers, core-shell, and mesoporous) that can be synthesized from different raw materials and their emerging applications in bioimaging, biosensing, drug delivery, tissue engineering, antimicrobial, and agro-foods. Depending on their morphology (e.g., size, aspect ratio, geometry, porosity), nanomaterials can be used as formulation modifiers, moisturizers, nanofillers, additives, membranes, and films. As toxicological assessment depends on sizes and morphologies, stringent regulation is needed from the testing of efficient nanomaterials dosages. The challenges and perspectives for an industrial breakthrough of nanomaterials are related to the optimization of production and processing conditions.

## 1. Introduction 

In the last 50 years, material researchers have been extensively studying how to exploit nanoparticles and nanostructured materials in different biomedical and healthcare sectors [1]. The term “NP” usually defines minute particles of matter (1 to 100 nm in diameter), but other names can be used to describe larger particles (up to 500 nm in diameter). For example, nanorods, nanowires, and nanofibers are nanoparticles with a diameter in the 1–100 nm range but with one dimension outside the nanoscale dimension [2]. Nanostructured materials are nanomaterials with one dimension in the nanoscale range (<100 nm) and are made of a single material or multiple materials. Therefore, nanostructured materials are composed of interlinked parts in the nanoscale range [3]. Nanoparticles and nanostructured materials can be made of simple materials (e.g., metal, carbon, polymer) [4], of composites (e.g., polymer-metal, silica-metal, graphene-metal), or in the core-shell form [5,6,7,8].

Nanomaterials are typically synthesized by one of two main approaches, i.e., bottom-up approach and top-down approach. Among all the methods, recently, the synthesis of nanomaterials by physical vapor deposition, chemical vapor deposition, electrospinning, 3D printing, biological synthesis, and supercritical fluid have gained importance, which is mingled with other methods to improve the synthesis efficiency [9,10]. Nanomaterials display many interesting features, such as superior mechanical performance, the possibility of surface functionalization, large surface area, and tunable porosity, compared to their bulk materials [11,12,13]. These outstanding features explain why nanomaterials are the perfect candidates in the biomedical sector for the production of tissue-engineered scaffolds (e.g., blood vessels, bone), drug delivery systems (gene therapy, cancer treatments, drugs for chronic respiratory infections), chemical sensors [4,5], biosensors [6,7], and wound dressings [14,15]. Remarkably, several studies suggest that ancient civilizations in India, Egypt, and China used nanotechnology (metallic gold) for therapeutic purposes in 2500 BC [16]. Nanomaterials’ discrete features can complicate the assessment of the effects and the toxicity risk associated with their use in a biological environment. Indeed, nanomaterials’ chemical composition, size, shape, surface charge, area, and entry route in the body can influence their biological activities and effects [17]. 

In bioimaging, tailored fluorescent nanoparticles could outperform traditional molecular probes as fluorescent indicators, particularly in terms of sensitivity [18]. Tissue-engineered nanofiber scaffolds are considered the best option to manage tissue loss and end-stage organ failure and have already helped millions of patients worldwide [15]. Three-dimensional nanofibrous scaffolds are polymer-based structures with balanced moisture, absorption, strongly organized porosity (60–90%), and gas permeability, comparable to native extracellular matrices [15]. One and two-dimensional nanomaterials can be used for signal amplification, are nanosized (≤100 nm), have high electrical conductivity, and are compatible with drugs [13] and biological molecules [12]. They have also been used for the early detection of diseases (e.g., virus, bacterial, cancer). Antimicrobial nanomaterials (e.g., Ag, Au, CuO NPs) are frequently employed in dermatology because they contribute to accelerating wound healing and preventing/treating bacterial infections [19,20].

Based on dimensionality, nanomaterials are classified mainly into four groups [1]: 0D, where length, height, and width are fixed at a single point; 1D, where only one parameter exists (e.g., carbon nanotubes); 2D, where length and width exist (e.g., graphene); and 3D, where length, height, and width exist [21]. For example, a graphene nanosheet is a typical example of a 2D nanostructure with a thickness in the nanoscale range. Theoretically, single-layer graphene is 0.345 nm thick (one atom thickness) and up to 500 nm in diameter. Based on their chemical composition, nanoparticles and nanostructured materials can be categorized into four types: organic nanomaterials (e.g., micelles, dendrimers, polymersomes, hydrogels, nanoconjugates), inorganic nanomaterials (e.g., metals, metal oxide, and ceramic nanomaterials), carbon-based nanomaterials (fullerenes, carbon nanofibers, diamonds carbon nanotubes, and graphene), and composite nanostructures [1,22]. The synthesis of traditional nanosized products contributes to the present and future economic growth of many countries. Based on porosity, nanomaterials can be classified into porous and non-porous materials [3]. Porous materials have a less dense molecular structure to allow airflow or the absorption of atoms, ions, and molecules. Non-porous materials are denser, do not absorb well, and allow limited airflow. Mesoporous (or super-nanoporous) nanomaterials are nanoporous materials with pores of 2–50 nm in diameter [23,24]. Recent research has focused on mesoporous nanomaterials for delivering therapeutic agents to tumor cells with little drug leakage into healthy cells. The high porosity, surface functionality, and small pore size of mesoporous nanoparticles allow the controlled release and efficient drug loading at the target site [22]. 

**Figure 1:**
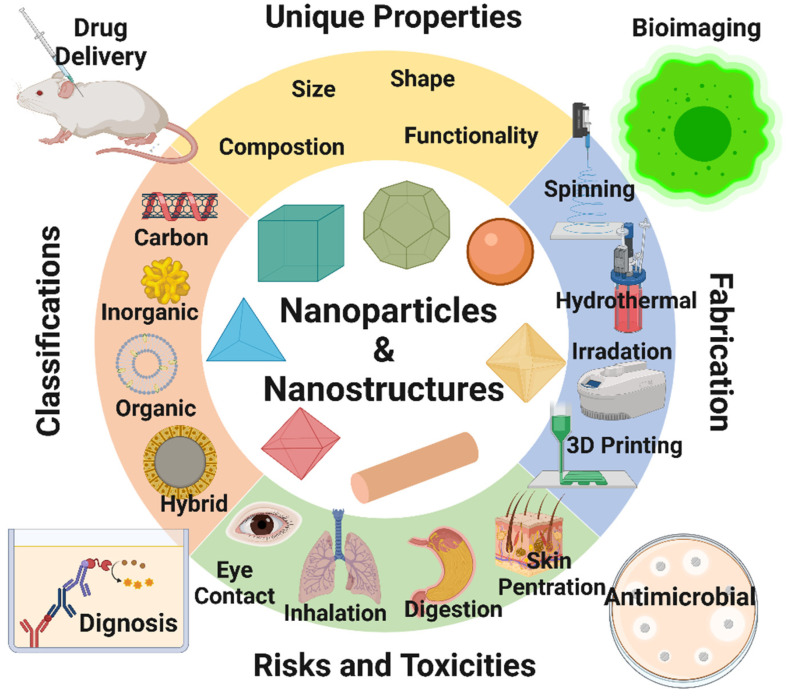
Summary of the recent topic on nanoparticles and nanostructured materials and their applications in bioimaging, biosensing, drug delivery, tissue engineering, antimicrobial, and agro-food sectors. Image created by Biorender.

Considering the growing use of nanomaterials over the last decades, our group reviewed the key aspects of different types of nanomaterial design and their emerging applications in the biomedical fields [1,2,3,15]. This review article discusses the unique features of nanomaterials that are exploited for different biomedical applications (Figure 1). It also presents recent trends on nanomaterials use for biomedical engineering, with a particular emphasis on the preparation methods used for designing nanomaterials. The advancement of nanomaterials in the biomedical and health sectors (bioimaging, tissue engineering, wound dressings, drug delivery, biosensors, food industry, and agriculture) is discussed. The review deals with various relevant nanomaterials variables, such as charge, concentration, particle surface modification, and size, which must be considered during the bioimaging of living cells. Various targeted drug delivery systems for treating chronic diseases are described. The use of nanomaterials to improve dental implants is also discussed, as well as the fabrication methods [14]. As many review articles have already described the biomedical applications of nanomaterials [1,2,3,4], this review will be extended to discuss the nanomaterial’s toxicity, risks, and regulations in the biomedical sector.

## 2. Fluorescent Nanomaterials for Bioimaging

**Figure 2:**
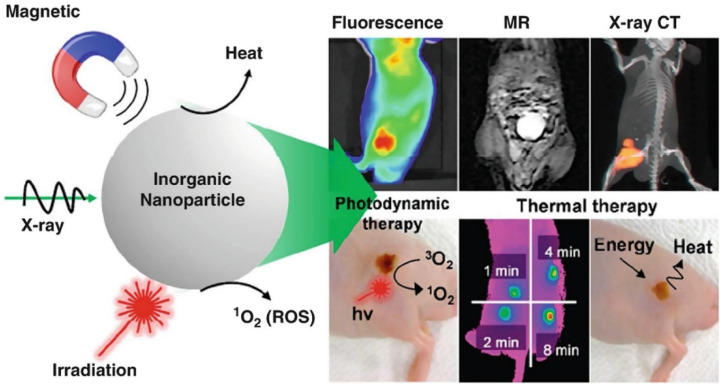
Schematic representation showing the utilization of magnetic nanoparticles in tumor bioimaging and therapy [30]. Copyright 2016, American Chemical Society.

Bioimaging is an advanced non-invasive technology used to visualize internal structures and physiological processes in living cells/organisms in real-time. It is a safe and effective technique for monitoring biological functions without affecting normal life processes (e.g., respiration and movement). It also helps to obtain data on the sample 3D nanostructure [25] and to investigate tissues at the subcellular and multicellular scale [26]. Several nanoprobes have been developed for bioimaging and the treatment of many diseases (cancer, heart, and inflammatory diseases) [27,28,29]. Nanomaterials are ideal materials for nanoprobes because they can be exactly characterized using nuclear magnetic resonance or gel permeation chromatography and are easily secreted from the body. However, their functions are limited, and researchers are always looking for new materials. In recent days, magnetic nanoparticles have gained great interest because of their progress in image-guided therapy (e.g., fluorescence, magnetic resonance, X-ray CT) and cancer theranosis favorable properties, such as tunable size, generating reactive oxygen species (ROS) or heat, simple fabrication, energy transfer, and X-ray absorption properties (Figure 2). Moreover, their long-term toxicity and dispersion stability must be specifically investigated.

Fluorescent nanoparticles’ specificity, light-emission (NIR-IR emission), and biocompatibility with the target tissues can be customized by changing their shape, size, and surface properties [27]. The cell uptake of nanoparticles used for bioimaging is influenced mainly by the following factors [31,32]: (i) size, smaller nanoparticles with identical surface properties are better absorbed than bigger nanoparticles [33]; (ii) charge, positively charged nanoparticles are preferentially taken up by living cells due to the cell membrane negative charge [34,35]; (iii) cell-specific targeting, this is achieved by conjugating nanoparticles with ligands that can interact with receptors at the cell surface [36,37]; (iv) the conjugation of proteins on the nanoparticles’ surface may promote rapid absorption and endosome bypass [38]; (v) oligodeoxynucleotide conjugation can help in the presence of complementary cellular DNA at a specific subcellular location [39,40]; (vi) endosome egress of nanoparticles that are positively charged at the surface in the low pH environment of late endosomes. It was reported that small nanoparticles can bypass the degradation pathways more efficiently than larger size particles with the same chemical composition [41].

**Figure 3:**
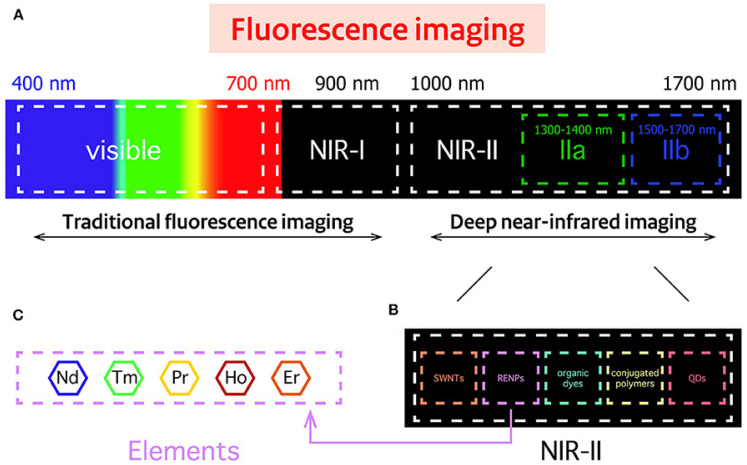
Schematic presentation showing fluorescence imaging approaches of traditional methods versus rare-earth-metal doped nanoparticles. (**A**) The spectral range of classical fluorescence imaging methods. NIR, near-infrared region. (**B**) Examples of probes in the NIR-II region: single-walled carbon nanotubes (SWNTs), rare-earth-metal doped nanoparticles (RENPs), organic dyes, conjugated polymers, and quantum dots (QDs). (**C**) Nanoparticles are doped with rare-earth metals (Nd, Tm, Pr, Ho, Er) [42]. Copyright 2020, Frontiers.

The fluorescent nanoparticles used in bioimaging can be classified into two categories: (1) fluorescent nanoparticles that can emit specific optical signals, such as carbon and metallic quantum dots, (2) fluorescent nanoparticles that require labeling with a fluorophore to be visualized, such as fluorescence mesoporous SiO_2_ and Fe_2_O_3_ NPs, liposomes, protein-based, and polymeric nanoparticles. Many classical fluorophores emit fluorescence at a short wavelength (e.g., the ultraviolet (UV) and visible regions) that can be easily absorbed and scattered by human tissues. This can lead to specific issues, for instance, elevated auto-fluorescence, low signal-to-background ratio, and limited tissue penetration. High-energy light could be used to overcome these drawbacks, but it can cause phototoxicity in human tissues. Optical imaging in the near-infrared (NIR) region is a possible alternative (Figure 3). Fluorophores in the first near-infrared window (NIR-I, 700–900 nm) have been tested and show good sensitivity; however, their application in bioimaging is limited due to their poor tissue penetration (less to 1 cm) and large photon scattering losses in biological matter. Therefore, fluorescent quantum dots (≤10 nm) that allow fluorescent bioimaging in the NIR-II window (1000–1700 nm) have been developed [42]. 

Fluorescent metal quantum dots (e.g., Au, ZnSe, InAs, CdTe, InP, or CdS) with a size in the 1–10 nm range show broad absorbance bands and narrow emission bands and are interesting for bioimaging in the NIR range [43]. These inorganic nanoparticles are the most frequently used in bioimaging because of their intense color, shape, size, and high-power surface photoluminescence. They allow the non-invasive detection of disease and the monitoring of its progression/response to treatments in humans and animals [44]. Metal oxide (Fe_3_O_4_, WO_3_, WO_2.9_) [30,32], lanthanide-doped nanoparticles [45], and quantum dots (QDs) [30] have also been tested for bioimaging and therapy. The nanoparticles optical and physical features can be tailored by structure amplification, which is their most distinctive advantage for optical imaging. Ceramic nanomaterials (mesoporous TiO_2_ and SiO_2_ NPs) also are among the most interesting candidates for bioimaging because of their size-manageable morphology, easy functionalization, and biocompatible hydrophilic surface [46,47]. FDA-approved silica is less toxic and is biocompatible [48]. Magnetic metal oxide nanoparticles can be exploited as nanocarriers for substances that are optically active or that can emit optical signals upon excitation, depending on their structural composition. However, using magnetic metal oxide nanoparticles in bioimaging is challenging due to their loading capability, synthesis complexity, regulatory hurdles, imaging efficiency, toxicity of the intrinsic ingredients, batch reproducibility, production cost, in vivo stability, and storage [49,50].

Fluorescent carbon nanoparticles are also interesting for bioimaging applications [51] for several reasons, particularly source abundance, simple synthesis, low cost, and non-toxicity [52]. Fluorescent carbon nanoparticles, such as fullerenes and carbon quantum dots, are considered to be promising nanomaterials alternatives to fluorescent semiconductor quantum dots, which are composed of toxic heavy metals, such as cadmium [53,54]. Fullerenes and carbon quantum dots are strong candidates for bioimaging applications [55]. Indeed, they are superior to the currently used inorganic quantum dots and traditional organic fluorophores in terms of photo-bleaching resistance, easy surface functionalization, and chemical inertness [56]. Their greater aqueous solubility, minimum cytotoxicity, and substantial fluorescence quantum yields explain their suitability for biomedical applications, specifically for in-vitro and in-vivo bioimaging [57,58].

Rare-earth metals are primarily used to develop nanoparticles with persistent luminescence [58]. However, the major issue linked to the use of rare-earth metal-based nanoparticles in vivo is their potential cytotoxicity and their low tunability for surface modification [59,60]. Therefore, non-toxic conjugated polymer nanoparticles with tremendous optical properties have been developed to act as persistent luminescence materials that can replace the conventional rare-earth metal-based nanoparticles. Conjugated polymer nanoparticles display excellent photoelectronic properties due to the highly localized π electrons on their backbones [61]. Additionally, due to their light-harvesting and light-amplifying properties, conjugated polymers act as biological photoactive materials. Water-soluble conjugated polymers have been developed and used in drug/gene delivery, biosensing, bioimaging, and antitumor/antimicrobial therapeutics [62,63]. Moreover, it has been demonstrated that conjugated polymers do not have any cytotoxic effect on the host cells and have structure-dependent tunable optical properties [64,65]. Currently, conjugated polymer nanoparticles can be prepared using four different approaches: solvent exchange (nanoprecipitation), mini-emulsion, self-assembly, and emulsion polymerization.

**Figure 4:**
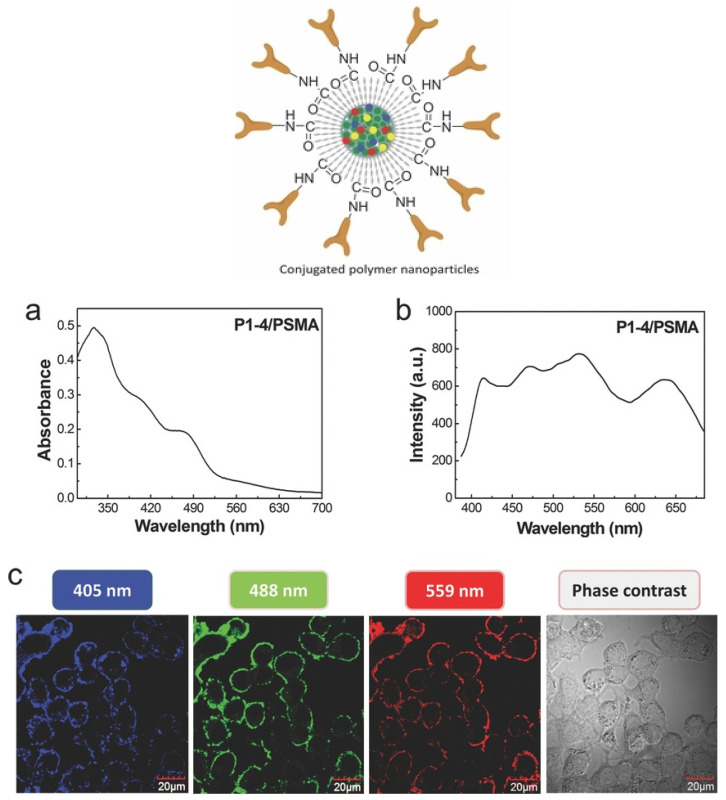
Multicolor conjugated polymer with carboxyl groups were fabricated from poly (styrene co-maleic anhydride) (PSMA) with four conjugated polymers (P1, P2, P3, and P4), for cancer cell bioimaging and detection. (**a**) UV-vis absorption, and (**b**) fluorescence emission spectra of P1–4/PSMA nanoparticles in water (excitation wavelength: 360 nm). The conjugated polymer nanoparticles were fabricated by precipitation of the tetrahydrofuran solution (2.0 µg/mL of P1, 7.0 µg/mL of P2, 4.0 µg/mL of P3, 12.0 µg/mL of P4, and 20.0 µg/mL of PSMA) into water. (**c**) Multi-channel fluorescence images of MCF-7 cells using P1–4/PSMA/anti-EpCAM polymer nanoparticles. The excitation wavelengths are indicated above the panels [67]. Copyright 2014, Wiley.

Du et al. [66] described a fast and multimodal approach for in vivo fluorescence bioimaging through the synthesis of Fe and Zn nanoclusters in HepG2, HeLa, and U87 cancer cells. They showed that fluorescent magnetic Fe_3_O_4_ nanoclusters and ZnO nanoclusters are synthesized by cancer cells in which Zn^2+^ and Fe^2+^ (which are biocompatible) have been introduced. These nanoclusters are promising candidates for multiplexed imaging that integrates fluorescence imaging, computed tomography, and magnetic resonance imaging. Moreover, they did not observe any significant variation in the fluorescence signal before and after the injection of Zn^2+^ and Fe^2+^ in normal human liver cells (L02) and tissues. This suggests that this multiplexed bioimaging system could be interesting for monitoring the response to cancer treatment and also for cancer diagnosis. Wang et al. manufactured multi-colored conjugated polymer nanoparticles for targeted tumor imaging (Figure 4) [67,68]. Functionalized conjugated polymer nanoparticles with carboxyl groups were fabricated by coprecipitation of poly (styrene co-maleic anhydride) (PSMA) with four conjugated polymers (P1, P2, P3, and P4) with blue, green, yellow, and red fluorescence emission, respectively [67]. Energy could be transferred from nanoparticles with shorter wavelength emission to nanoparticles with longer wavelength emission, leading to the formation of multi-colored nanoparticles with whole visible light absorption and emission spectrum via only one excitation. For enhancing their specificity towards tumor cells, conjugated polymers (P1-4/PSMA) were labeled with an antibody against EpCAM (a protein overexpressed in many cancer types). Excitation was recorded in antibody-labeled conjugated polymers (P1-4/PSMA/anti-EpCAM) at different wavelengths. Then, due to the antibody cross-reactivity, Wang et al. used P3/PSMA/anti-EpCAM and P3/PSMA/anti-ErbB2 to differentiate between HeLa and MCF-7 cells (EpCAM), and SK-BR-3 cells (ErbB2), although MCF-7 and SK-BR-3 cells were derived from the same patient [67,69].

Understanding biological causes and developing therapeutic strategies requires the ability to track cell migration and circulation in vivo. Many attempts have been undertaken to investigate novel and improved cell labeling and imaging techniques, and much has already been learned from these approaches. The capacity to visualize these cells precisely could help in the diagnosis and prognosis of disorders, such as atherosclerosis, neurodegenerative diseases, cancer, and myocardial infarction. Understanding the behavior of circulating cell populations, such as monocytes, macrophages, and circulating tumor cells (CTCs) spread from solid tumors, could provide crucial information on features that could aid in the development of better treatments [70]. Chen’s team observed CTC behavior in blood arteries surrounding solid tumors using a multiphoton device with a 30 Hz collection rate. The highest number of circulating tumor cells each minute was estimated to be around 100, which could be linked to cancer progression [71]. Furthermore, by labeling circulating tumor cells with antibody-conjugated quantum dots, the behavior of uncommon subpopulations of circulating tumor cells with higher metastatic potential, such as CD24+ and CD133+ CTCs, has been observed. Jia’s team produced doxorubicin (DOX)-loaded MSNs that are decorated with anti-EpCAM and anti-CD44 aptamers to deliver cancer metastasis chemopreventive medications selectively toward circulating tumor cells. DOX-fluorescence MSNs enable the self-tracking of targeting efficacy and drug administration into colorectal cancer cells. It was possible to track not only how the transplanted CD45-CD541CD1571 lung stem/progenitor cells were precisely located in vivo but also how these cells incorporated and reformed themselves over time at a single-cell resolution using a combination of fluorescent nanodiamonds, fluorescence-activated cell sorting, and fluorescence lifetime imaging microscopy. HNF3 plasmid DNA (pDNA) was efficiently transported by fluorescein isothiocyanate (FITC)-tagged MSNs as a differentiating agent for iPSCs. The indoctrination procedure was simultaneously observed due to their self-monitoring capacity [72].

Surface functionalization of nanoparticles is a crucial issue for their use in bioimaging applications (Table 1) [68,69]. However, the selection of the most suitable nanoparticles for a specific bioimaging application (e.g., computed tomography) is difficult, and several aspects (biological safety, sensor capacity, size, brightness, and photostability) must be taken into account. After finding the best material for a specific application, all experimental methods must be adapted and adjusted to the selected material. This is not an easy task. A deeper knowledge of the impact of nanoparticles on living cells will help to understand the imaging potential of those nanoparticles and how it is adsorbed by cells. Therefore, more research efforts are needed to promote nanoparticle exploitation in bioimaging applications.

## 3. Nano-Drug Delivery Systems

**Figure 5:**
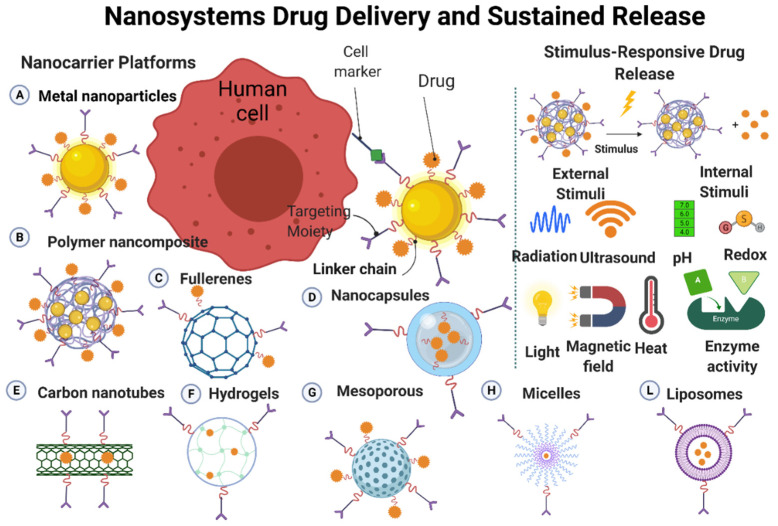
Characteristics of nanomaterials that can cross the biological membranes to deliver a drug to a specific site and mechanisms influencing controlled drug release. Image created by Biorender.

Drug delivery systems are quite new, but is a rapidly expanding technology. In these systems, nanoscale materials are used to deliver the therapeutically active drug or the imaging molecule (when used as diagnostic tools) to the targets [83]. Nanostructures (made of metals, organic/inorganic, and polymeric materials) are often used for the development of drug delivery systems (Figure 5). Nanoparticles and nanostructured materials are crucial components in these carrier systems that play a key role in personalized medicine by improving drug formulation/targeting/controlled release [83,84]. Such systems can deliver a drug to a specific site at a predetermined rate and in a predesigned manner; consequently, the drug bioavailability will be enhanced, while side effects will be reduced. Drugs can be physically or chemically adsorbed into the nanoparticles surface through various adsorption methods, or they can be loaded on nanoparticles during their production [83,85]. The drug and carrier properties, such as drug-carrier solubility, molecular weight, drug-carrier chemical interaction, and carrier size, will determine the drug loading efficacy on/into the carrier [83,86]. The drug release rate from the nanoparticlesis mainly influenced by (1) the release of the adsorbed drug from the surface of the nanoparticles; (2) the drug diffusion from thenanoparticles; and (3) the nanoparticle erosion and drug diffusion from the nanoparticles. Therefore, the drug release rate from the nanoparticles will be governed by polymer biodegradation and drug diffusion. The drug release time and location can be modulated by the nanoparticles composition (e.g., thermosensitive and pH-sensitive materials) and engineering (e.g., monolayer and multilayer nanoparticles, nanocapsules), and also by better understanding the physiological factors involved in this process [83,87] (Figure 5).

Many researchers worldwide are investigating whether and how nanoparticles (e.g., metals, metal oxides, carbon, quantum dots, liposomes, dendrimers) can be used as carriers for different therapeutic agents [88,89]. Graphene oxide nanosheets, graphene quantum dots, single/multiwalled carbon nanotubes, and graphene oxide nanosheets are carbon-based nanomaterials with different drug-loading capacities, targeting specificity, and drug release kinetics. This partly explains the discrepancies in the therapeutic efficiency of these different nanomaterials when used as drug-carrier systems. Biodegradable polymer nanoparticles are used in new drug delivery systems because of their flexibility and many interesting characteristics, such as controlled release, stability in blood, non-immunogenic, and non-toxic nature [85]. Micelles, liposomes, emulsions, and nanoparticles are colloidal drug carriers that are used to increase the number of drugs that can pass through the blood–brain barrier. Similarly, colloidal systems are used to regulate the drug release rate at target locations (cells or tissues).

Drug delivery via nanocarrier systems presents some strengths compared with conventional drug administration methods, particularly very high accuracy, targeting ability, stability, and sustainability at the target location [83,90]. Delivery systems made of large-size materials display major drawbacks, such as poor absorption, in vivo stability, bioavailability, and solubility, which may decrease their efficacy and target specificity. The major goal of using nanoparticles as drug carriers is to reduce the drug toxicity and increase its bioavailability, target specificity, and delivery without any loss of the therapeutic effects [83,91]. The key challenges when looking for suitable carriers are: (1) drug release kinetics and integration, (2) shelf life and stability of the formulation, (3) biocompatibility of the formulation, (4) biodistribution and targeting, and (5) possible nanocarrier accumulation in the body in the case of prolonged treatment using drug-loaded nanocarriers. Currently, the available data do not allow the determination of the toxicological and environmental impacts of drug-loaded nanocarriers [83,92]. Furthermore, drugs can also be synthesized at the nanoscale, and then they can act as their own “carrier” for delivery. Table 2 lists different nanocarriers used for the delivery of therapeutic molecules/drugs.

### 3.1. Nano-Vehicles for Anticancer Drugs

After heart disease, cancer is the second most common cause of death worldwide [109,110]. Functionalized nanocarriers can be used to develop targeted anticancer treatments. Compared with systemic chemotherapy, in these systems, nanoparticles loaded with therapeutics and targeting molecules are specifically delivered to the tumor cells and show good efficacy at lower doses and, consequently, with fewer undesirable effects [111]. Several studies suggest that nanocarriers are interesting tools to improve cancer diagnosis (as imaging agents) and treatment [112,113]. Over the last two decades, nanoparticles products have been evaluated in several clinical trials [114].

The efficacy of chemotherapeutic agents at the target site can be increased by encapsulating them in nanoparticles that target the tumor cells actively or passively. Nanoparticles offer various advantages when used for drug delivery: (i) overcoming the stability and solubility issues of chemotherapeutic drugs; (ii) protecting the drug from modifications by enzymes (e.g., proteases and other metabolic enzymes) and, thus, also increasing the drug half-life in blood; (iii) increasing drug targeting and distribution; (iv) modulating the drug release kinetics; and (v) reducing resistance to treatment by delivering multiple drugs [84].

Often, after reaching the target, the anticancer drug efficacy is significantly reduced for many different reasons (e.g., too low concentration) [84]. As the therapeutic effect of a drug is possible only if present at the right concentration and in the correct form, nanoparticles used as carriers could increase the local drug concentration inside and around tumor cells. This also decreases the risk of toxicity for healthy cells. The drug-carrying nanoparticles deliver the drug directly into its targeted body area (organ, cellular, and subcellular level of specific tissue) to overcome the specific toxic effect of conventional drug delivery, thereby reducing the amount of drug required for therapeutic efficacy. As a result, the use of nanoparticles in drug delivery opens new possibilities for improving drug distribution and changing cancer management [115]. The interaction of nanoparticles with ligands (nucleic acid aptamers, peptides, antibodies, carbon dioxide, and tiny molecules) may contribute to the active targeting of cancer cells and organs.

Polymeric nanoparticles can be used for the controlled release of the encapsulated drugs by surface erosion, diffusion, and swelling, followed by diffusion, depending on the time and condition. The most widely used biocompatible polymers for controlling drug release are poly(D, L-lactide), poly(glycolide), and its co-polymer poly(lactic-co-glycolic acid) [116]. Biodegradable polymer nanoparticles for cancer treatment have been extensively studied [117,118]. Liposomes were the first nanoparticles to be used to administer chemotherapy. Cohen and Bangham were the first to describe liposomes 40 years ago [119]. Liposomes are structured like vesicles with an aqueous interior and one or more concentrically phospholipid bilayers with diameters ranging from 30 nm to several microns. They are produced using different methods and display different sizes, lipid compositions, and surface chemistry. Liposomes can be used as flexible carriers that can be tailored to various functions and have a specific drug delivery role [120]. Several materials, such as biodegradable polymers, dendrimers, and nucleic acid-based nanoparticles, have also been used to develop targeted cancer treatments [121,122].

Electrospun nanofibers have also been evaluated for localized anticancer drug delivery [113,114]. They can efficiently deliver the desired drug to the target cancer cells compared with conventional diffusion-based drug delivery vehicles [122,123,124,125]. Zhang et al. manufactured a nanofiber-based localized anticancer drug delivery system by electrospinning a solution containing self-assembled PEG_2000_-Pt(IV) micelles with dichloroacetate (DCA) that “acts as a prodrug” and with polyvinyl alcohol. This delivery system allowed the quick release of PEG_2000_-Pt(IV)-based micelles and DCA that showed a synergistic apoptotic effect on cancer cells through two apoptotic mechanisms [107]. Table 3 lists various nanomaterial types exploited in delivery systems for anticancer agents.

### 3.2. Nanostructured Materials as Drug Delivery Vehicles for Antioxidant Drugs

Antioxidants are reactive molecules that are produced by the body in response to environmental stress and other stimuli and that contribute to limiting cell damage by free radicals. Therefore, they are also called “free-radical scavengers”. Antioxidants are present in many food types and can also be synthesized. However, due to their limited cellular absorption, potency, and lack of precise transportation systems to a specific organ, cell, and tissue, their use for treating diseases in which oxidative stress plays a major role is still limited, particularly for neurodegenerative diseases where the brain targeting is still challenging [83]. Nanotechnology can address these drawbacks, especially the targeting of dietary antioxidants with neuroprotective properties. Indeed, antioxidant molecules can be protected from degradation using nanotechnology-based delivery mechanisms that improve their bioavailability and physicochemical drug-like properties [134].

However, most nanoparticle-based formulations tend to cause oxidative stress in the cells, and this hampers their routine clinical use. Importantly, the amount of reactive oxygen species (ROS) produced by the cells is proportional to the concentration of nanoparticles to which the cells were exposed [135]. Moreover, the cellular redox balance is influenced by nanomaterials, and this can lead to the inhibition or induction of ROS production [83]. Excess reactive oxygen species production and endogenous antioxidant system overload are common side effects of high nanoparticle concentrations, resulting in cytotoxicity and inflammation [83,136]. As a result, determining the maximum permissible doses is important to minimize negative effects. However, several studies suggest that low nanoparticle exposure levels can unintentionally boost the antioxidant defenses and reduce oxidative stress. Moreover, some nanomaterials have enzyme-like antioxidant properties, and they reduce oxidative damage and scavenge reactive oxygen species and free radicals [137].

**Figure 6:**
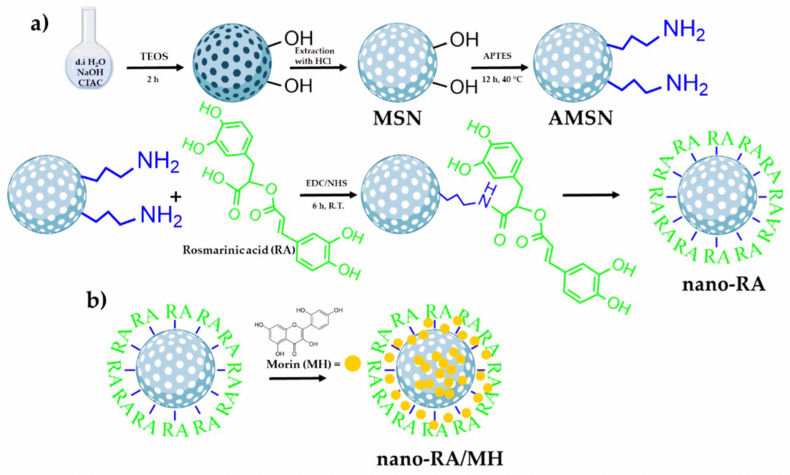
Schematic presentation showing the preparation of morin-loaded nano-antioxidants of (nano-RA/MH) loaded onto mesoporous silica nanoparticles (MSN). (**a**) The graphic path from the single components to therosmarinic acid nanocarrier (nano-RA) and (**b**) morin loading on the nanocarrier (nano-RA/MH) [149]. Copyright 2016, MDPI.

Nanoparticles’ antioxidant properties depend on their surface load, volume surface ratio, chemical composition, particle size, and surface coating [138]. Nanoparticles offer many advantages compared with traditional methods of antioxidant supplementation, including the environmental safety of bioactive materials, improved bioavailability and selective antioxidant supplementation, and controlled release at the target site [139,140]. The antioxidant function of transition metal oxide nanoparticles (CuO, NiO NPs) has been widely studied and exploited [141,142,143,144]. Cerium oxide nanoparticles (CeO_2_ NPs) are particularly interesting because of their reactive oxygen species (ROS) scavenging and regenerative effects [145]. These nanoparticles display special features: the coexistence in both oxidation states (Ce^3+^ and Ce^4+^), reversible switching between these states, and reduction potential of 1.52 V [146]. Gold nanoparticles (Au NPs) have been extensively evaluated by the pharmacology and biomedical sectors due to their inert and non-toxic nature [147]. Silver nanoparticles (Ag NPs) also have a strong antioxidant capacity (reduction power and free-radical scavenging) [148]. Arriagada et al. [149] prepared antioxidant mesoporous SiO_2_ nanoparticles on porous nanoplatforms with rosmarinic acid (nano-RA) as an antioxidant that was loaded on the mesoporous SiO_2_ nanoparticle surface. Morin flavonoids were incorporated on the antioxidant nanocarrier by the impregnation/solvent evaporation technique with high drug loading (23% wt/wt) compared to bare mesoporous SiO_2_ NPs (9% *w*/*w*). In addition, the rosmarinic acid and mesoporous SiO_2_ nanoparticle (nano-RA) release profile was evaluated using two biorelevant media. The antioxidant activity of the rosmarinic acid and mesoporous SiO_2_ nanoparticles (nano-RA) was maintained, suggesting the correct disposition of the moiety (Figure 6) [149]. These results suggest a promising antioxidant nanocarrier suitable for future application in drug delivery [149]. Table 4 lists different nanomaterial types used as carriers for antioxidant delivery.

## 4. Antimicrobial Materials

Antibacterial agents are used in the biomedical sector, textile industry, water treatment, and food industries [165]. The antimicrobial characteristics of nanoparticles are influenced by several factors [166], including size, shape, and the type of encapsulated antibiotics drug. For instance, nanoparticles with angular shapes (e.g., triangular, cubic, tetrahedral) cause mechanical harm to the microbial membrane, and this contributes to increased microbial growth inhibition compared to spherical nanoparticles [167]. Based on their antimicrobial properties, antimicrobial nanoparticles can be classified into four main categories: antibacterial, antifungal, antiviral, and antiparasitic nanoparticles [168]. Table 5 lists these different types of antimicrobial nanoparticles, their mode of action, and targeted microorganisms. For millennia, metals and metallic salts have been used for their antibacterial properties. For instance, silver pots have been used for drinking water since 4000 BCE [169].

Surface functionalization allows the production of antibacterial nanoparticles with two different antibiotics to concomitantly kill, for instance, Gram-positive (encapsulated) and Gram-negative (attached) bacterial strains. Inorganic disinfectants, such as metal oxide nanoparticles, are gaining popularity because of the limitations of organic disinfectants, such as toxicity to humans [166,170]. Currently, nanophysics researchers are investigating the effects of different metallic nanoparticles in bacteria. The antibacterial mechanism of metallic nanoparticles is still debated, but three major mechanisms are proposed: (i) reactive oxygen species (ROS) formation; (ii) metal ion release from metallic nanoparticles; and (iii) metallic nanoparticles interaction with the cell membrane. Metallic nanoparticles display higher antibacterial effects than their salts. The antibacterial function is often influenced by the size of the metallic nanoparticles [20].

**Figure 7:**
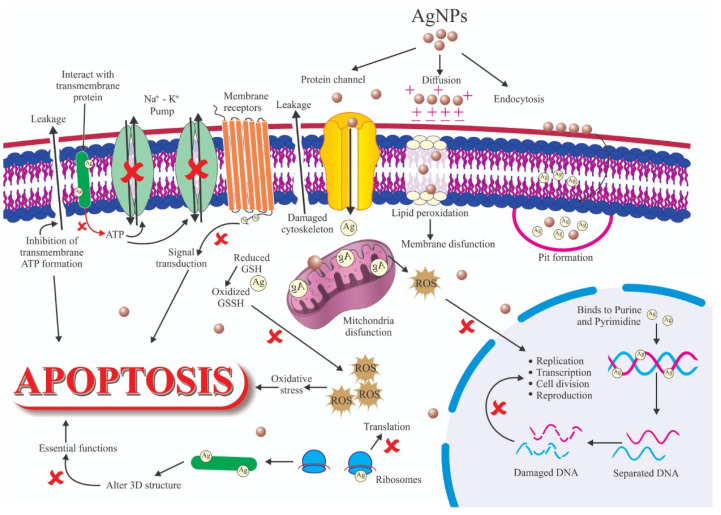
Schematic representation of several factors that influence silver nanoparticles’ (Ag NPs) antibacterial activity.

The antimicrobial properties of silver nanoparticles (Ag NPs) are very well known, and when compared to other metallic nanoparticles, they have higher toxicity against microorganisms [198,199]. As they are currently used as an alternative to antibiotic treatment, Ag NPs are often referred to as “next-generation antibiotics”. The antimicrobial potential of Ag NPs has been tested in many different pathogens, including Gram-(+)/(−) bacteria, viruses, and fungi. These particles also show antimicrobial activity in many multidrug-resistant bacteria [200,201]. Despite the many studies on Ag NPs, the precise mechanism of their antimicrobial effect is still unknown [202]. It is thought that their antibacterial activity relies mainly on the generation of Ag^+^ ions. Some studies showed that Ag^+^ ion generation is influenced by the nanoparticle’s surface area. Specifically, nanoparticles with a larger surface area generate higher Ag^+^ concentrations, which leads to enhanced antimicrobial activity. Conversely, nanoparticles with a smaller surface area generate lower Ag^+^ concentrations and thus display lower antimicrobial activity [203]. This also indicates that the antibacterial activity is linked to the amount and quality of Ag^+^ ions generated. In addition, Ag NPs’ toxic effects against bacteria are influenced by the nanoparticle’s physicochemical properties. Many studies have shown that Ag NPs disrupt several metabolic pathways and cellular pathways inside the bacterial cell [35,204,205]. The antibacterial activity of Ag NPs is influenced by several factors related to the nanoparticles (size, shape, coating) and the medium (light, oxidative species, ligands, ionic strength). The mechanisms whereby these factors can modify the antibacterial activity of Ag NPs are many (Figure 7) and include ligand replacement, oxidative dissolution, Ag^+^ ions reduction, passivation of the Ag surface, passivation layer puncturing, silver speciation, and nanoparticle aggregation [206]. These phenomena may also be influenced by some chemical species. For instance, chloride can accelerate or slow down corrosion in the function of its concentration. Therefore, the antimicrobial activity of Ag NPs should be assessed in controlled conditions to limit/prevent unexpected changes in the system. Specifically, Ag NPs must be stored in the dark and without oxygen. Antibacterial mechanisms can be classified in two groups: non-oxidative and oxidative mechanisms (Figure 7).

The antimicrobial and antibiofilm properties of TiO_2_ NPs are widely known and are active against pathogens, such as fungi, viruses, parasites, and bacteria. Recently, the use of TiO_2_ NPs in the food industry has started and has been approved by the FDA for their use in drugs, food, and cosmetics [207]. The quantum size effects and photocatalytic effects of these NPs made them ideal in various antimicrobial applications, such as antimicrobial coatings on medical devices, air, and water purification. Anti Gram-positive and Gram-negative effects of TiO_2_ NPs are well known, but the bactericidal activity of these NPs has been enhanced by plant extracts, such as Garcinia zeylanica. The combination of inherent antimicrobial activity with plant extract and photocatalytic property of TiO_2_ NPs have enhanced their potency as microbicidal agents [208]. ROS generated by TiO_2_ NPs destruct the microorganisms by oxidizing the cell membrane. Apart from antimicrobial activity due to photocatalytic activity TiO_2_ NPs, these nanoparticles also show activity in the absence of light by direct contact and adsorption of cells and cause the loss of membrane integrity [209,210]. The antimicrobial properties of Cu NPs are widely known on various species of bacteria (e.g., bacillus subtilis, methicillin-resistant staphylococcus aureus, Pseudomonas aeruginosa, salmonella choleraesuis). The level of agglomeration of Cu NPs decides the microbicidal activity, which is a common issue with them. Sammler-sized Cu NPs result from a reduction in the agglomeration, which increases the surface area and interaction with bacterial membranes that lead to more toxicity. Hydroxyl radicals were produced from the ionic and metallic forms of copper that damage essential DNA and proteins [211,212]. Many other nanoparticles, such as Si NPs, CaO NPs, MgO NPs, and Al_2_O_3_ NPs, have good antimicrobial properties with good biocompatibility. Most of them will act by damaging the bacterial cell wall [212].

## 5. Gene Therapy

Gene therapy is a method in which genes are modified to prevent and/or treat disease. With this revolutionary technique, clinicians can treat a disease simply by incorporating the modified gene into the patient’s cells, without the need for surgery or drugs. Depending on the disorder, gene therapy may be used to add a functional copy of a gene that is not working properly or to switch off the gene that is causing the problem. These modified genes are delivered into the cells using a vector (i.e., a genetically modified transporter). Due to their nanometric size, large surface-to-volume ratio, and stability, nanoparticles are attractive agents as gene carriers [83].

Surface modifications may be used to bind an infinite number of ligands and receptors [207]. Alternatively, nanoparticles may encapsulate and release nucleic acids into target cells, with superior efficacy in gene therapy compared with non-viral vectors and without immunogenicity. Liposomes have been studied as medication and also as a DNA delivery device [208]. For instance, gene therapy in which liposomes act as vectors has been used to treat corneal diseases [209], cardiovascular diseases [210], cystic fibrosis [211], and cancer [212]. In lung carcinoma, cancer with low survival rate, DOTAP:cholesterol liposomes were used to deliver the tumor suppressor gene FUS1 in mice harboring lung cancer xenografts [213]. Liposome–DNA complexes were synthesized using a simple mixing method. The complex efficacy in inhibiting tumor growth and metastasis development was evaluated [37].

**Figure 8:**
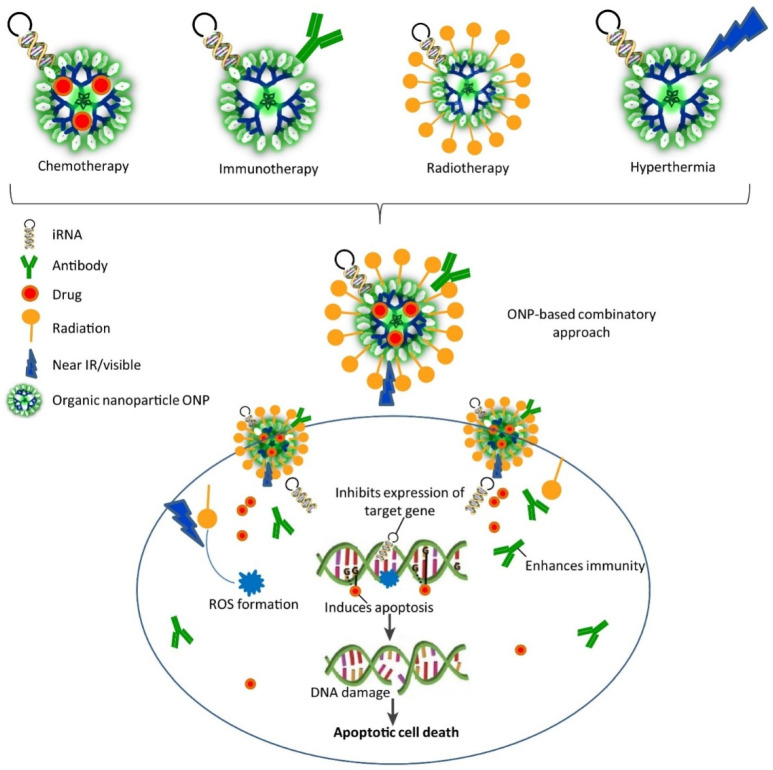
Combinatorial approaches based on organic nanoparticles (ONP) for gene therapy are associated with other therapies [214]. Copyright 2017, Trends in Biotechnology.

Designing nanoparticles for gene delivery is not easy because many factors must be taken into account. First, nanoparticle functionalization should bring biocompatible layers and contribute to maintaining the structural integrity and activity of the transported genes/drugs in the biological fluids. Second, nanoparticle production, like for any therapeutic product, must take into account the following issues: physicochemical properties, biopharmaceutical properties, and pharmacological properties. Therefore, nanoparticles should have the capacity to treat directly, and this requires new design parameters. Studies on the Administration, Distribution, Metabolism, and Excretion (ADME) of nanoparticles should be designed to take into account their aggregation and surface characteristics (Figure 8) [120].

In plant science, gene delivery plays a vital part in the growth of new plant varieties and the development of drought-resistant, pest-resistant, high-yield plants [215]. The main inherent obstacle to gene delivery in plants is the biomolecule movement within cells through the rigid and multilayered cell wall. Many delivery methods have several disadvantages, including low production, contamination, and foreign DNA incorporation into the plant genome [83,216]. The functionalization of biomaterials allows the development of bionanomaterials to deliver a gene into plant cells through a nanoneedle that can overcome the existing limitations in delivering biomolecules to plants [217]. Intracellular gene delivery has been performed using conventional synthetic vehicles, such as cationic lipids, dendrimers, and polymers. It safeguards DNA from nuclear enzyme degradation, ensures efficient movement inside the cells and tissues, as well as the active gene transfer to the cell nucleus. Mesoporous SiO_2_ nanoparticles have also been tested to deliver genetic material to plant cells. Traditional methods of gene transfer have some drawbacks, such as the limited amount of delivered DNA, cell degradation, limited plant diversity, and toxicity; however, new vehicles for activators, nucleic acids, and proteins into vegetable cells are now available showing higher efficiency and additional safety features [218].

## 6. Biosensors

**Figure 9:**
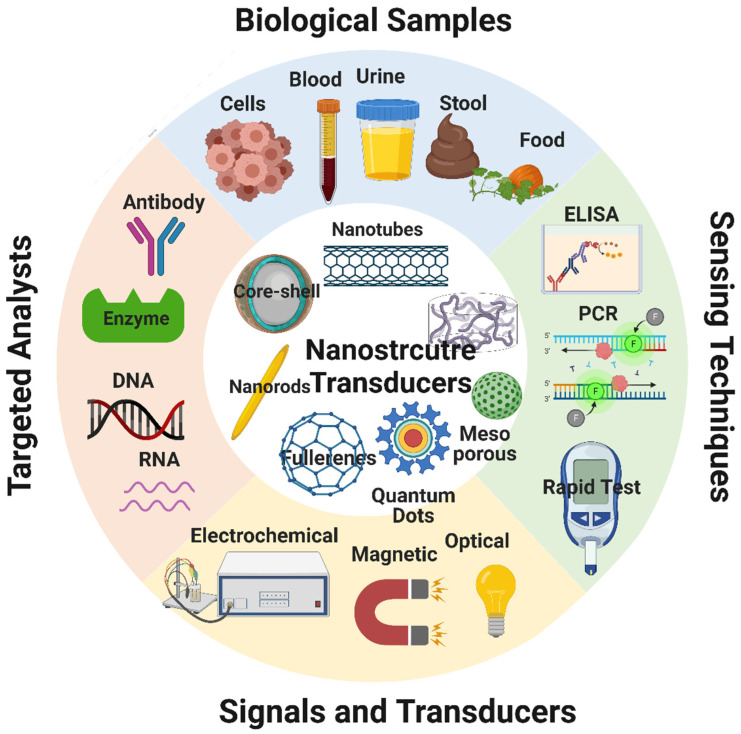
Schematic representation of the components of a typical biosensor and of the different types of bioreceptors and transducers. Image created by Biorender.

Biosensors are devices that combine organic components (e.g., antibodies, enzymes) and an electronic element to yield a detectable signal that can be quantified [219]. The electronic elements detect the physiological change produced by the interaction of the organic component with environmental chemical or biological elements [220,221]. A typical biosensor consists of five main components (Figure 9): (i) the analyte, a substance the presence and amount of which are detected [222]; (ii) the receptor, an organic molecule that can detect the analyte [221]; (iii) the transducer, a device that converts the physiological change occurring following the analyte-receptor interaction to a quantitatively measurable optical or electrical signal [220]; (iv) the electronic part that receives and quantifies the signals from the transducer; and (v) the display, an interpretation system (a computer and a printer) to display the response output in a manner that can be understood by the user [223].

The efficient signal collection is one of the main challenges in biosensor development (transduction). The interaction of the analyte with the biological element is converted into gravimetric, electrochemical, magnetic, electro-chemiluminescent, or optical signals using a transducer. Engineered nanomaterials have a greater electrical conductivity, are nanosized, may be used to amplify desired signals, and are biocompatible. Due to their potential to trap huge amounts of specific binding units and to operate as a conductive medium, nanomaterials are good candidates to improve the biosensor detection sensitivity for specific molecules. Carbon nanotubes, nanodiamonds, semiconductor quantum dots, polymer nanofibers, and graphene are some of the most studied nanomaterials for biosensing applications. Indeed, carbon compounds can be used to conjugate biomolecules (enzymes, antibodies, DNA, cells). Nanomaterials can improve biosensor performance (better sensitivity and lower limit of detection). Nanomaterials with optimal surface-to-volume ratio, chemical activity, mechanical strength, electrocatalytic capabilities, and diffusivity can profoundly influence biosensor performance. Moreover, nanomaterials biocompatibility is a key feature in building biosensors to monitor bacteria, viruses, DNA, and other biomolecules [224].

Nanomaterial-based biosensors have been used for diagnostic purposes through the detection of specific biomarkers in biological samples. For example, Tang et al. manufactured a complex biosensor for the detection of prostate cancer biomarkers by using magnetic nanoparticles as a carrier for the labels and as separators. Specifically, the magnetic nanoparticles used for the amperometry detection of four prostate cancer biomarkers (interleukin-6, prostate-specific antigen, platelet factor-4, and prostate-specific membrane antigen) were labeled with horseradish peroxidase (HRP) and secondary antibodies (Ab2) to yield Ab2-MNP-HRP beads. Then, beads were resuspended in a phosphate-based buffer and incubated with a mixture of protein standards to detect the four markers within this mixture. Afterward, the mixture was transferred to the sensing compartment labeled with primary antibodies against the four markers under which ring-shaped magnets were fixed to facilitate the bead migration to the sensing surface. Following the incubation and washing steps, the addition of the HRP substrate enabled the development of a detectable and measurable electrochemical signal [225]. Table 6 lists the benefits of various nanomaterials.

## 7. Tissue Engineering

Tissue engineering is a multidisciplinary approach to develop structures that are made of biological components (e.g., cells, stimulatory molecules) and biomaterials that can mimic the native organ/tissue. Engineered tissues may be used at the place of conventional organ/tissue transplant procedures to lower the cost burden [230,231]. The rapidly evolving nanotechnological and fabrication techniques have allowed the incorporation of various biocompatible nanomaterials in tissue engineering, including nanoporous scaffolds and nanofiber membranes [232,233]. Nanomaterials have been used in various tissue engineering applications (periodontal, neural, bone, and skin tissue engineering) [234]. Nanomaterials can contribute to finely tuning the scaffold characteristics, particularly the mechanical strength, and regulating the release of bioactive molecules (growth factors, cytokines, inhibitors, genes, drugs) [235,236].

**Figure 10:**
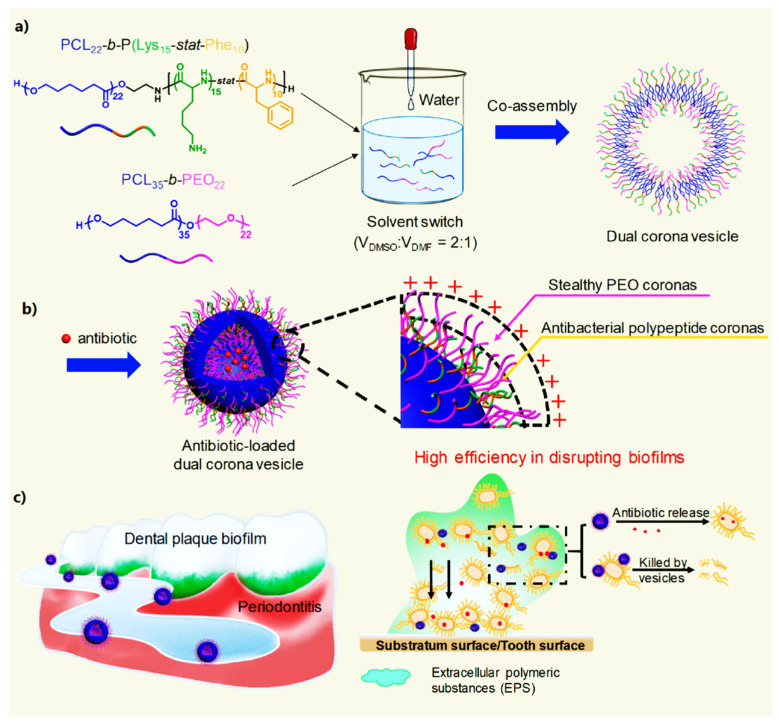
Vesicles displaying antibacterial activity and good antibiotic delivery capacity for the management of biofilm-induced periodontitis. (**a**) Co-assemblage of multifunctional corona vesicles. (**b**) Encapsulation of ciprofloxacin within the multifunctional corona vesicles. (**c**) Antibacterial activity of the multifunctional corona vesicles to remove dental plaque biofilms produced by bacteria [238]. Copyright 2019, American Chemical Society.

In dental tissue engineering, novel treatments are required for the effective reconstruction of periodontal tissue damage due to the gradual loss of the self-healing capacities of periodontal tissue with age. Nanomaterials have emerged as promising candidates for the reconstruction of periodontal tissue [237]. Nanomaterials are interesting materials in dental tissue engineering as (i) nano-coatings for dental implants, (ii) nanofillers to enhance the mechanical properties of the biomaterials used in dental tissue engineering, (iii) antimicrobial agents to prevent oral infections, and (iv) as ingredients for novel personal care products and toothpaste [237]. Xi et al. prepared multifunctional vesicles by co-assembling poly(ε-caprolactone)-block-poly(lysine-stat-phenylalanine) and poly(ethylene oxide)-block-poly(ε-caprolactone) that were loaded with ciprofloxacin hydrochloride (an antibiotic used to treat periodontitis) (Figure 10). Their in vitro and in vivo experiments showed that these multifunctional vesicles eliminated biofilms made by Escherichia coli and Staphylococcus aureus and greatly contributed to periodontitis treatment [238].

In humans, the nervous system is an extremely complex system that comprises the peripheral nervous system (PNS, motor, and sensory nerves) and the central nervous system (CNS, spinal cord, and the brain). The nervous system lacks self-healing capacities; therefore, any trauma or disease-related damage to the CNS or PNS is permanent [239]. The frequency of neurological damage increases with age and is becoming a public health issue due to the aging population [240]. Currently, protocols of neurological damage treatment are based on autologous or allogeneic cell grafts and neurosurgery. However, these approaches often require the inhibition of the immune response (grafts) and additional surgical procedures and show moderate effects [241].

**Figure 11:**
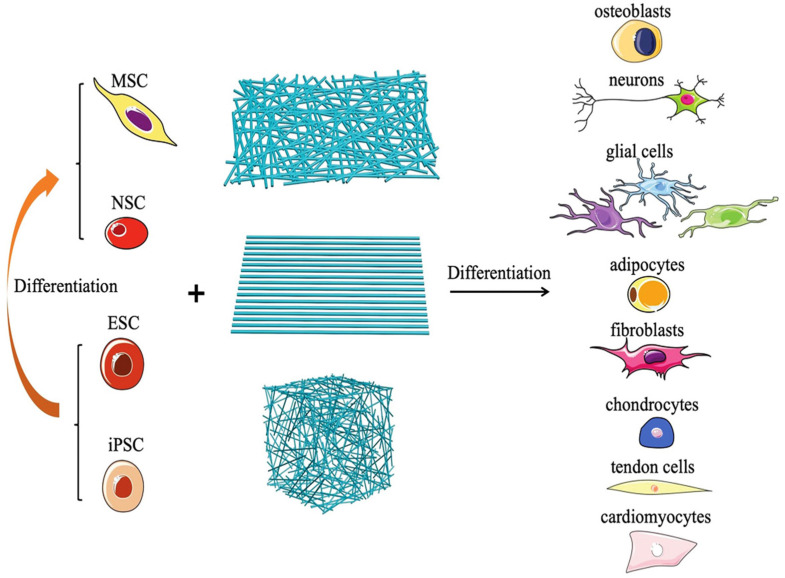
Schematic figure to show how electrospun nanofibers promote the differentiation of various types of pluripotent stem cells into different lineages [248]. Copyright 2020, Wiely.

Nanofibers were initially defined as fibers with diameters below 100 nm. However, the scope of nanofibers has been broadened in recent years, with all fibers of diameter less than 1 μm included. Several techniques have been reported to prepare nanofibers, including the splitting of bicomponent fibers, melt-blowing, physical drawing, flash-spinning, phase separation, self-assembling, solvent dispersion, centrifugal spinning, hydrothermal, and electrospinning [242,243,244,245]. Nanofibers present diverse applications, such as molecular filters, bioseparation, bio-sensing, crop protection, bioremediation, anti-counterfeiting, and antibacterial. The high surface area to weight ratio of nanofibers makes an ideal substrate for molecular filtration and shows potential for forming a scaffold for protective clothing applications against biochemical attacks [246,247]. Nanofibrous materials are gaining interest for tissue engineering applications, including skin regeneration. Nanofibrous structures mimic the native extracellular matrix and promote the adhesion of various cells and soluble factors that may promote cell function and tissue regeneration (Figure 11) [248].

Wound healing is a natural process that involves hemostasis, inflammation at the site of injury, the proliferation of keratinocytes, and remodeling [249]. Based on the healing time, skin wounds are categorized in chronic and acute wounds [250]. Compared with acute wounds, chronic wounds need much more time to heal, and they are mainly observed in people with comorbidities (e.g., diabetes, obesity) [251]. The formation of a new blood vessel (angiogenesis) is crucial for the wound healing process because it is required for the flow of nutrients, waste, and oxygen to the wound site. It also accelerates the rate of granulation tissue formation. Any angiogenesis impairment will result in a chronic wound [252]. Therefore, during the treatment of a skin wound, the successful formation of the new blood supply must be taken into consideration. Currently, various protocols are available for chronic wound management, such as ozone therapy, hyperbaric oxygen therapy, oxygen therapy, and negative pressure wound therapy [253]. Moreover, for large skin wounds, autologous skin grafts are considered the gold standard. However, their use is limited by the small amount of donor tissue that can be obtained and by the morbidity at the donor site [254].

Recently, nanomaterials have been proposed as candidates for nanostructured scaffold architectures for the management of large skin wounds. This is explained mainly by their distinct physicochemical features, particularly their nanoscale dimensions and their very high surface-area-to-volume ratio. Nanomaterials can also be used in skin tissue engineering as delivery vehicles of therapeutic molecules [19]. Randeria et al. prepared Au NPs functionalized with small interfering RNAs against ganglioside-mono sialic acid 3 synthase (GM3S), thiolated ethylene glycol, and dispersed in Aquaphor. GM3S is an enzyme the expression of which is increased in diabetic mice and that causes insulin resistance, thus slowing down wound healing. The authors found that in diabetic mice, these functionalized Au NPs could downregulate GM3S expression and that skin wounds in nanoparticle-treated mice fully healed in 12 days compared with untreated mice [255].

## 8. Agriculture and Food Industry

Nanotechnology is used in agriculture to enhance food production and also to improve/preserve the nutritional content, quality, and safety of foods. Fertilizers, insecticides, herbicides, and plant growth factors/regulators are used to enhance agricultural yields. Nanotechnology is also more and more implicated in the development of approaches to stimulate seed germination, plant growth, and plant defenses [256]. Metal nanoparticles (Ag NPs and Cu NPs) have been particularly studied in plant science. Their organic synthesis is very expensive and involves dangerous chemicals [257]; however, nanoparticle surface functionalization allows the accommodation of more micronutrients in one nanoparticle for efficient delivery to the plants. These micronutrients may enhance productivity and increase the nutrient content in agriproducts. Carbon nanomaterials are commonly used in agriculture because they can influence the plant’s metabolic functions, and ultimately, its growth. Therefore, these nanomaterials, used at very small concentrations, can go into the plant cells and could be an effective answer to increase crop yield and fruit production [258].

The plant’s response to nanomaterials is influenced by different factors, including the nanoparticle’s size, shape, application process, and chemical and physical properties. Nanomaterials can be used as nanostructured fertilizers to increase the absorption and efficiency of traditional fertilizers (nutrients and phosphates). It has been shown that soybean growth and seed yield are increased by 33% and 20%, respectively, when treated with hydroxyapatite nanoparticles (phosphorous nano-fertilizers) compared with normal phosphorous fertilizers. Moreover, nano-fertilizers are used at lower concentrations, thus limiting the nutrient spreading to runoff or groundwater and reducing the risks of degradation and toxic effects due to over-application [259]. Mung bean, cucumber, and rapeseed plants have been treated with ZnO, Fe_2_O_3_, TiO_2_, and CuONPs, as nano-fertilizers, by direct addition to the soil or through irrigation or foliar application [260,261]. In tomato cultures, soil supplements have doubled the number of flowers and fruits, probably by activating plant-based genes/proteins [262].

**Figure 12:**
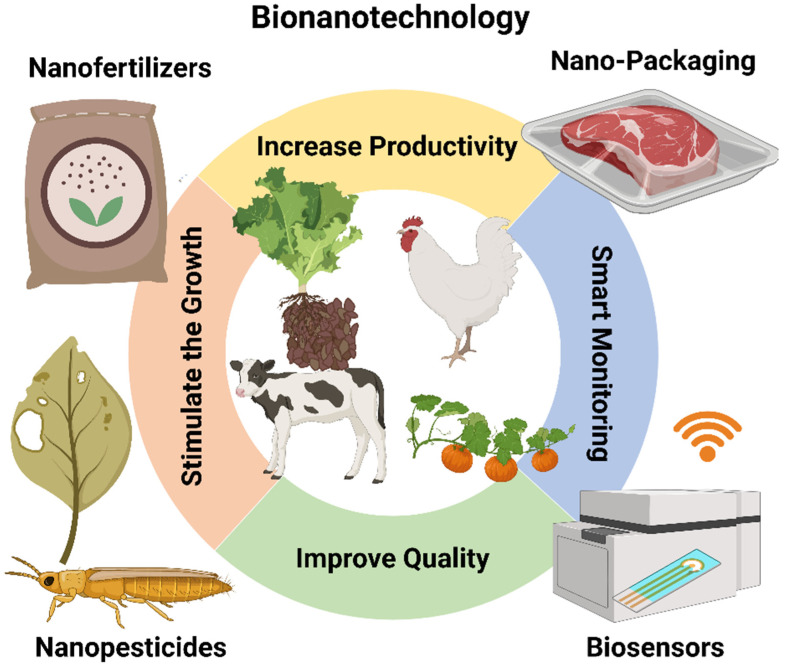
Potential applications of nanomaterials in the animal and agriculture industry. Increase the productivity of the crop using nano-pesticides and smart packaging; Improve the quality of the soil using nano-fertilizers; Stimulate animal and plant growth using nanomaterials; Provide smart monitoring for animals and plants using nanosensors by wireless communication devices. Image created by Biorender.

In wheat cultures, chitosan nanoparticles have been used to manage the release of nitrogen, phosphorus, and potassium via foliar intake [263]. In terms of environmental degradation, organic nanoparticles are more appropriate. However, their nutrient supply advantages compared with conventional fertilizers need to be more rigorously demonstrated [259]. Microgel-based fertilizers biofunctionalized for the delivery of the micronutrients to the plant have been developed for foliar delivery of nutrients [264]. Nano-fertilizers distributed in a controlled manner can boost crop development, yield, and productivity. Crop enhancement can also be obtained by gene transfer using nano-based target delivery approaches. Nano-pesticides can protect crops effectively. Precision farming is considerably promoted by the development of nanosensors and digital controls. Plant drought tolerance and soil enrichment can both benefit from nanomaterials. Figure 12 shows some potential applications of nanomaterials in the animals and agriculture industry, i.e., nano-fertilizers and nano-pesticides, which stimulate animal and plant growth using nanomaterials; smart monitoring for animals and plants using nanosensors by wireless communication devices and smart packaging.

Fertilizers significantly increase farm productivity. However, as their over use irreversibly changes the soil chemistry, nanomaterial-based fertilizers are more frequently proposed to increase the fertilizer uptake by plants and to minimize the amount of fertilizer needed, and thus, its concentration in the soil. Sustainable farming implies minimal use of chemicals to protect the environment and various plant/animal species against extinction [265]. Nanomaterials can boost crop growth/yield by facilitating the fertilizer uptake by the plant, thus ensuring minimal use of inputs and encouraging site-oriented, regulated nutrient supply. Indeed, nanotechnology research (and funding) on plant protection has been growing to guarantee better crop yields. Many plants exposed to toxic concentrations of metal ions and nanoparticles attempt to prevent or reduce uptake into root cells by restricting metal ions (nanoparticles) to the apoplast, binding them to the cell wall or cellular exudates, or by inhibiting long-distance transport [266].

Through effective farming, irrigation, and utilization of quality seeds, agricultural yields can be improved by 35–40%. The use of nano-formulation fertilizers strongly improves crop productivity. Carbon nanoparticles in fertilizers, for example, increase the yield of rice (by 10.29%), soybean (by 16.74%), winter wheat (by 28.81%), vegetables (by 12.34–19.76%), and spring maize (by 10.93%). Nanomaterials are the best strategy to bring nutrients to plants that are extremely porous at the nanoscale through the activation of different biological plant processes. As a consequence, nano-fertilizers may increase the absorption of nutrients through the plant pores [267]. Furthermore, extensive research has clearly shown that reducing the nanomaterials size increases the surface mass of particles, thus adsorbing and slowly and steadily desorbing a vast amount of nutrient ions over a long period [268]. Surface-modified nanomaterials-based fertilizers can accommodate more than one nutrient into the same carrier. Thus, nano-fertilizer formulations enhance the growth of nutritionally healthy crops and increase yields. It should be remembered that improved crop production will push farmers to increase the use of that product (Figure 12) [269].

To protect the crops from diseases caused by fungi and other plant pathogens and to increase productivity, pesticides and herbicides can be encapsulated into surface-functionalized nanoparticles. The use of this nano-formulation type has increased exponentially. Traditional crop management practices involve the large-scale use of fungicides, herbicides, and insecticides at large concentrations. Nanotechnology has been extensively employed to regulate the application of phytopathogens, and nano-fungicides and nano-pesticides are now commonly used in agriculture [270]. Encapsulation in nanoparticles allows the gradual and regulated release of the pesticide active ingredients, often leading to the decrease of pesticide utilization and, consequently, of their emissions into the atmosphere. The nanosensor-based detection of pathogens may also reduce the risk of disease in addition to nano-pesticides [265].

Fast-Moving Consumer Goods (FMCG) are fast-selling consumer items, such as household cleaning materials, toiletries, cosmetics, pharmaceuticals, and also include many non-sustainable items, such as batteries, glassware, and light bulbs. However, the main segment consists of food and beverage items that have a limited shelf life, either because of strong market demand or because the commodity depreciates rapidly or is perishable. Therefore, the packaging of such products is crucial to reduce waste and to avoid product damage during its transport to stores and the consumers’ houses [271]. Nanotechnology research in the food industry is focused on the development of techniques for food manufacturing, packaging, and distribution. Many food items are now supplemented with nanoparticles that enhance nutrient and bioactive distribution mechanisms, taste and texture, and microbiological safety. In the field of food production and labeling, nanoparticles are also used as antimicrobials or as extremely reactive biosensors for the identification of bacteria, allergens, pollutants, and degradants that may influence food quality and health [272]. Therefore, today, many food products contain nanoparticles (intentionally introduced or due to contamination). Nanomaterial pollution may also originate in the agricultural environment where many nanomaterials are used (pesticides and fertilizers, cattle protection, poultry development) [273]. Table 7 summaries the nanomaterial applications in the agri-food sector

## 9. Risks of Exposure to Nanomaterials

**Figure 13:**
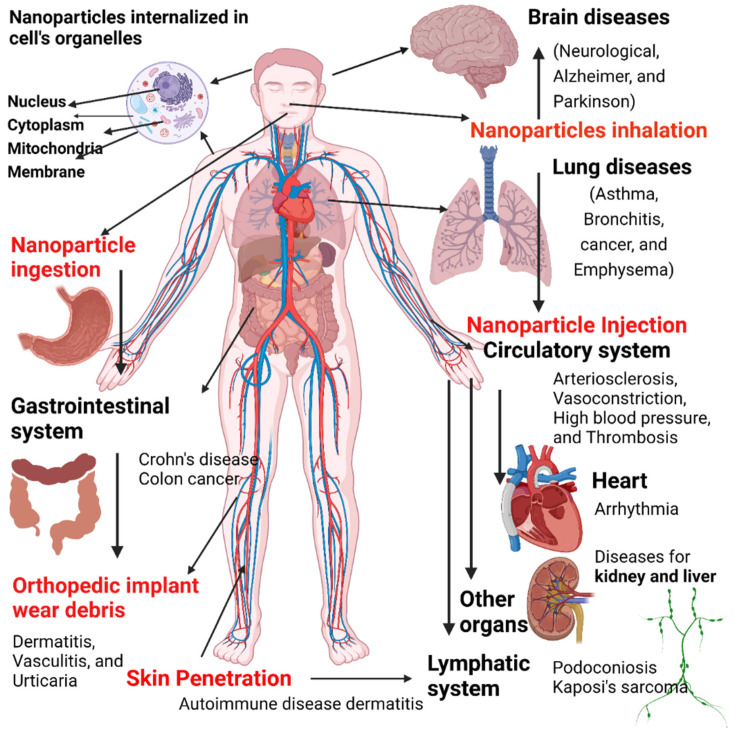
Disease caused by exposure to nanoparticles and entrances of nanoscale materials into the body through inhalation, dermal exposure, and ingestion, resulting in many potential hazards. Image created by Biorender.

Despite their advantages, nanomaterials may also be associated with risk factors. Nanoparticles can adversely affect different organs/tissues in the body and can be associated with different disorders (Figure 13). Nanoparticle exposure can promote the development of neurological disorders (e.g., Parkinson’s disease and Alzheimer’s disease), lung (asthma, bronchitis, emphysema, and cancer), and cardiovascular diseases (atherosclerosis, arrhythmia, thrombosis, and hypertension). In addition, exposure to nanoparticles can cause skin irritation, dermatitis, urticaria, and other skin problems.

Various factors influence the toxicity of the different nanomaterials, particularly the exposure time and dose. The exposure dose can be determined by multiplying the molar concentration of nanoparticles in the medium by the exposure time [63]. However, other factors (e.g., aggregation and concentration effects) may also influence the nanoparticle’s toxicity. For instance, some nanoparticles can aggregate. These aggregates (in the micrometer range) might not easily penetrate the body, and thus their toxicity is decreased. Toxicity is also influenced by the nanoparticle’s size because nanoparticles with a size of ~10 nm can go through the cell membrane, and thus, can be more toxic than larger nanoparticles [288]. Nanoparticles’ shape also influences their toxicity, which may vary in function of the aspect ratio. For instance, 10 µm asbestos fibers cause lung cancer, whereas smaller fibers induce mesothelioma and asbestosis [289]. Similarly, the nanoparticle effect is inversely proportional to the nanoparticles size and directly proportional to the surface area. The cell uptake, subcellular localization, and oxidative mechanisms can also be influenced by the crystal structure [287]. For example, a comparison of two polymorphous structures of TiO_2_ NPs showed that one causes DNA damage through oxidation but not the other [290]. The nanoparticle’s surface properties can also contribute to their toxic effects through translocation and oxidation processes [291,292]. Table 8 summarizes the risk of toxicity of different nanoparticles.

## 10. Global Market and Future of Nanomaterials

Nanotechnology has evolved as a solution to many unsolved fundamental biological problems in the field of medicine, healthcare, and human life. This technology helps in the identification of various pathogens, toxins, pesticides, imaging of cancers, and also helps in the transport of active medicaments to the target sites effectively. Currently, many research institutes are involved in nanosystem research. Some of the outcomes of these studies are approved by internal nanotechnology organizations (e.g., the National Center for Nanoscience and Technology and the U.S. Food and Drug Administration) and are commercialized for their better applications in the fields mentioned above, and some of them are in the investigational stage. The biomedical application segment led the market and accounted for the largest revenue share of around 29.98% in 2020. The global nanomaterials market is expected to reach USD 57,608.26 million by 2026, growing at a CAGR of 19.86% during the forecast period (2021–2026). This growth is attributed to the wide range of applications of nanomaterials in the biomedical sector, including imaging, targeted drug delivery, nanorobots for surgery, nanodiagnostics, cell repair, and nanobiosensors [1,2,3,4].

The importance of nanotechnology and nanosciences open a wider scope for new biomedical-based applications using newly discovered nanomaterials. However, this growth in the use of nanomaterials will be limited by several challenges, including: (1) the ability to deliver cost-effective production of nanomaterials in large volumes; (2) defined production techniques that can be scaled up sufficiently to cover the cost required for targeting volume markets; (3) the rapid identification of priorities in nanoresearch to guarantee nanotechnologies’ and nanomaterial’s safety in the future; (4) address the gaps for current research in risk/toxicity/safety assessment for nanomaterials; and (5) develop an international standard for nanomaterial’s safety; to assist in the determination of appropriate risk management of nanomaterials [325,326,327].

The new and future innovation is nanomaterials that have exceptionally extraordinary properties in the food source chain (enhancement of food texture and quality, bioavailability, nutrient values, nano-pesticides, nano-fertilizers, and nano-herbicides) in the world agricultural sector. A large proportion of nanomedicines fail to meet such criteria, and as a result, they are not available in the pharmaceutical sector. Additionally, the scale-up and reproducibility of nanomedicines will be a future issue in formulation development. In the coming decade, nanomaterials will create a new history in the medical field by advancing new nanomaterials for their sustainable development. Synthesis of newer nanomaterials in the near future will revolutionize the treatment strategies for many diseases. There is a lot to achieve in the future in nanomaterial research for their extensive applications in various fields, such as biomedicine (bioimaging, biosensor), agriculture, environmental protection, and food processing.

## 11. Conclusions

Nanomaterials are interesting materials because of their superior and tunable physical, chemical, and biological features compared with bulk materials. Nanomaterials can be classified in function of their size, shape, composition, and origin. Researchers have exploited nanomaterial features by grafting different groups on them, thus making nanoparticles suitable for biomedical applications. In this review, we presented the applications of nanomaterials in bioimaging, skincare, tissue-engineered scaffolds, drug delivery systems, biosensors, and wound healing, as well as in the food and agricultural industries. The use of nanomaterials for targeted drug delivery has also dramatically progressed with exceptional applications to reduce the limitations of conventional drug delivery systems. Different nanomaterial types (e.g., spherical nanoparticles, core-shell, nanorods, nanowires, hollow, nanofibers, and mesoporous) are studied for the targeted delivery of drugs. Encapsulation techniques have also been tested to deliver various bioactive cytotoxic agents. Nanoparticles also have their place in tissue reengineering for the repair of various tissues. Thanks to their larger surface-area-to-volume ratio, nanostructured scaffolds can act as selective substrates to absorb specific proteins and promote cell adhesion. Carbon and metal-based nanoparticles for biosensor development can lead to many applications in agriculture and in the biomedical sector. The review also presented the fluorescence nanoparticles and the in vivo and in vitro fate of nanoparticles. The applications of fluorescence nanomaterials in bioimaging were explained clearly. The antimicrobial activities of various nanomaterials and their advantages and disadvantages are also discussed individually.

## Figures and Tables

**Table 1:** Different nanomaterial types are used for bioimaging applications.

Nanomaterial	Functionalization	Cell Lines	Refs
Graphene-based nanosheets	Surface functionalization by bio-compatible targeting ligands and coatings	MDA-MB-468 (MCF-7)	[70]
Molybdenum disulfide nanosheets	Chitosan; PLGA, PEG functionalization	Breast cancer cells (MDA-MB-468), HeLa uterine cancer cells, human lung cancer cells	[71]
Transition metal nanoparticles decorated with polymers	Polymer functionalization	Mice bearing 4T1 breast cancer cell xenografts	[72]
Lanthanide-activated nanoparticles	Doping with lanthanide	Cancer cells xenografted in mice	[73]
Group IV quantum dots	Surface functionalization	Various cancer cell types	[74]
Graphene oxide nanosheets	Surface functionalization	Tumor cells	[75]
Peptide-based nanoparticles	Chemical functionalization	Peptide-treated HeLa cells preloaded with Hg^2+^	[76]
Silver nanoparticles	Aptamer conjugation	Leukemia cells, neural stem cells, kidney tissue, renal carcinoma cells	[77]
Gold nanoprisms	Conjugation with polyethylene glycol	Gastrointestinal carcinoma cells (HT 29)	[78]
Gold nanorods	Encasing by mesoporous silica	Carcinoma cells	[79]
Magnetofluroscentnanoprobe	Surface functionalization	Human Breast Cancer (MCF-7), HeLa cells	[80]
Dye-loaded nanoemulsions	Lipids conjugation with polyethylene glycol	Human colon cancer (HCT116), HeLa cells	[81]
Cadmium telluride quantum dots	Capping by shells	Human bronchial epithelial cells	[82]

**Table 2:** Nanomaterials used for drug delivery.

Nanocarrier	Loaded Drug	Therapeutic Action	Ref
Metal-based nanoparticles			
Gold nanoparticles	Doxorubicin	Anticancer effect in HeLa cells	[93]
Gold nanoparticles	Theophylline (THP), 1,3-dipropyl-8- cyclopentylxanthine (DPCPX)	Neuron reconstruction in vivo	[94]
Silver nanoparticles	Methotrexate-coated PEG	Anticancer effect in MCF-7 cells	[95]
Metal oxide-based nanoparticles			
Fe_3_O_4_ nanoparticles	Doxorubicin	Anticancer effect in HeGP2 and Lo2 cells.	[96]
Fe_3_O_4_ nanoparticles	Fluorouracil	Anticancer effect in MCF-7 cells	[97]
Carbon-based nanoparticles			
Multilayer carbon nanotubes	Dexamethasone	Anti-inflammatory effect in Highly-Aggressively Proliferating Immortalized cells (HAPI)	[98]
Single-layer carbon nanotubes	Cisplatin	Anticancer effect in head and neck squamous carcinoma in vivo and in vitro	[99]
Quantum dots			
Ag–In–Zn–S quantum dots modified with 11-mercaptoundecanoic acid, L-cysteine, lipoic acid, and decorated with folic acid	Doxorubicin	Anticancer effect in A549 cells (human alveolar basal epithelial cells)	[100]
Nano-clays			
Laponite nanoplates	Anionic dexamethasone	Anti-inflammatory effect in MG-63 osteoblast-like cells	[101]
Dendrimers			
Poly-amido-amine dendrimers	Methotrexate	Anticancer effect in methotrexate -sensitive and resistant human acute lymphoblastoid leukemia (CCRF-CEM) and Chinese hamster ovary (CHO) cells	[102]
Polymeric nanoparticles			
Poly-lactic acid	Paclitaxel	Anticancer effect in a mouse model of ovarian cancer in vivo.	[103]
Chitosan	Tacrine	Therapeutic effect in a rat model of Alzheimer’s disease in vivo (preclinical study)	[104]
Liposomes			
Liposomes	Dexamethasone phosphate	Anti-inflammatory effect in a rat model of adjuvant-induced arthritis in vivo.	[105]
Liposomes	Cetuximab and oxaliplatin	Anticancer effect in mice xenografted with colon cancer cells in vivo	[106]
Nanofibers			
Polyvinyl alcohol	PEG_2000_-Pt(IV) micelles and dichloroacetate	Anticancer effect in mice xenografted with cervical cancer cells in vivo	[107]
Polylactic acid electrospun nanofibers	Doxorubicin	Anticancer effect in mice with secondary hepatic carcinoma in vivo	[108]

**Table 3:** Different examples for nanomaterials being used in anticancer drug delivery systems.

Nanomaterial	Anticancer Drug	Targeted Cancer Cells	Refs
Silver nanoparticles	Terminaliachebula	Breast cancer cells (MCF-7)	[126]
Glycerylmonooleate nanostructures	Doxorubicin hydrochloride	Breast cancer cells (MCF-7, MDA-MB-231)	[127]
Poly (3HB-co-4HB) biodegradable nanoparticles	Docetaxel	Breast and prostate cancer cells	[128]
Carbon nanodots	Irinotecan	Breast cancer cells (MCF-7, MDA-MB-231)	[129]
Polysaccharide nanoparticles	Lapatinib	Breast cancer cells (MCF-7/ADR)	[130]
Fe_3_O_4_ nanoparticles	Doxorubicin	HepGP2 liver cancer cells and LO2 liver cells	[96]
Fe_3_O_4_ nanoparticles	Fluorouracil	Tumor cells and in vitro analysis	[97]
Porous silicon nanoparticles	Doxorubicin and siRNA	Prostate cancer cells	[131]
Thermosensitiveliposomes coated with cetuximab	Doxorubicin	EGFR-expressing breast cancer cells	[132]
Iron oxide nanoparticles	Cetuximab	A431 (epidermoid carcinoma) cell lines	[133]

**Table 4:** Different nanomaterials are used as carriers for antioxidant delivery.

Nanomaterial	Antioxidant Agent	Applications	Refs
Conjugates	Superoxide dismutase	Superoxide conversion to hydrogen peroxide	[150]
Conjugates	Superoxide dismutase	Enhancing drug delivery to the brain	[151]
Conjugates	Catalases	Hydrogen peroxide conversion to water	[152]
Nanozymes	Catalases	Hydrogen peroxide conversion to water	[153]
GSH-PEGDA oligomer nanoparticle	Glutathione peroxidase	Reduction of lipid hydroperoxides and conversion of hydrogen peroxides to water	[154]
Liposomes	Vitamins	ROS scavenging and upregulation of antioxidant molecules	[155]
Solid lipid nanoparticles	Carotenoids	Singlet oxygen quenching, formation of provitamin A carotenoids (free radical scavengers)	[156]
Liposomes	Lycopene	Singlet oxygen quenching, formation of provitamin A carotenoids (free radical scavengers)	[155]
Liposomes	Polyphenol flavonoid catechins	Free-radical scavengers, carcinogenic activity, inhibition of proinflammatory kinases	[157]
Quercetin nanosuspensions	Quecetin	Protection against LDL oxidation	[157]
Silica nanoparticles	Gallic acid	Rapid H-atom transfer to diphenyl picryl hydrazine	[158]
Silica nanoparticles	3,4-di-tert-butyl-4- hydroxybenzoic acid	Improved thermal and oxidative stability of low-density polyethylene (LDPE) composites	[159]
PEG-coated silver nanoparticles	Salvianolic acid	Improved reactive oxygen species (ROS) scavenging and antioxidant activity in living cells	[160]
Mesoporous silica nanoparticles	Poly-tannic acid	Good antioxidant activity	[161]
Mesoporous silica nanoparticles	Morin	Potent quencher of singlet molecular oxygen (1O2), HO· scavenger	[162]
Ceria nanoparticles	Polyethylene glycol (PEG)-dendron phospholipids	Biocompatibility, reduction of cytotoxicity and oxidative stress	[163]
PLGA-PEG	Curcumin	Neuroprotection	[164]

**Table 5:** Classification of antimicrobial nanomaterials based on their antimicrobial properties, their different modes of action, and targeted microorganisms.

Function	Mode of Action	Nanomaterial	Target Microorganism	Ref
Antibacterial	Interaction with DNA, resulting in DNA replication inhibition ROS production Interaction with sulfur-containing proteins, leading to the inhibition of the activity of several enzymes	Silver nanoparticles (Ag NPs)	*Bacillus subtilis*	[171]
			*Staphylococcus aureus*	[172]
			*Methicillin-resistant coagulase-negative*	[173]
			*staphylococci*	[174]
		Titanium oxide nanoparticles (TiO_2_ NPs)	*Escherichia coli* 0157:H7	[175]
			*Staphylococcus aureus*	[176]
			*Pseudomonas fluorescens*	[177]
		Copper oxide nanoparticles (CuO NPs)	*Bacillus subtilis*	[178]
			*Listeria monocytogenes*	[179]
Antifungal	Disruption of the cell membrane integrity	Titanium oxide nanoparticles (TiO_2_ NPs)	*Candida* spp.	[180]
			*Penicillium expansum*	[181]
			*Aspergillus niger* spp.	[182]
			*Penicillium oxalicum*	[183]
		Siver nanoparticles (Ag NPs)	*Candida* spp.	[184]
		Magnesium oxide nanoparticles (MgO NPs)	*Saccharomyces cerevisiae*	[185]
			*Candida albicans*	[186]
Antiviral	Inhibition of virus attachment to the host cell membrane	Gold nanoparticles (Au NPs)	*Human immunodeficiency* virus	[187]
			*Influenza* virus	[188]
		Silver nanoparticles (Ag NPs)	*Herpes simplex* virus	[189]
			*Respiratory syncytial* virus	[190]
		Titanium oxide nanoparticles (TiO_2_ NPs)	Inactivation of *bacteriophages*	[191]
			Inactivation of Qβ and T4 *bacteriophages*	[192]
Antiparasitic	Inhibition of promastigote proliferation and metabolic activity Generation of ROS that may inhibit parasitic infection	Silver nanoparticles (Ag NPs)	*Leishmania tropica*	[193]
			*Leishmania infantum*	[194]
			*Entamoeba histolytica*	[195]
		Copper oxide nanoparticles (CuO NPs)	*Entamoeba histolytica*	[196]
			*Cryptosporidium parvum*	[197]

**Table 6:** Examples of different nanomaterials and their key benefits in various applications.

Nanomaterial	Dimentionality	Key Benefits	Ref
Spherical metallic nanoparticles	Zero-dimensional (0D)	Immobilization of bio-receptors Improved analyte loading Strong catalytic characteristics	[226]
Spherical quantum dots	Zero-dimensional (0D)	Excellent fluorescence, Charge carrier quantum confinement size-adjusted band energy	[227]
Nanorods	One-dimensional (1D)	Excellent plasmonic materials Size-adjustable energy regulation to produce specific field responses	[228]
Nanowires (1D)	One-dimensional (1D)	Superior charge conduction Strong sensing characteristics	[228]
Carbon nanomaterials (1D and 2D)	One and two-dimensional (1D and 2D)	Superior charge conduction High functionalization potential	[229]

**Table 7:** Summary of nanotechnology and nanomaterials applications in agri-food sector.

Agriculture	Food Processing	Food Packaging	Supplements	References
Detection of the specific molecule to estimate the enzyme-substrate interaction	Nanoencapsulation for bioavailability enhancement of nutraceuticals	Detection of foodborne chemicals and pathogens by fluorescent nanoparticles attached to antibodies	Nutrient absorption enhancement by nanosized powders	[274,275,276,277]
Delivery of pesticides and fertilizers through nanocapsules	Flavor enhancement using nanoencapsulation	Monitoring of temperature, moisture, and time using nanosensors	Cellulose nanocrystals function as drug carrier	[278,279,280,281]
Controlled delivery of growth hormones	Nanoparticles used as viscosifying agents	Ethylene detection by electrochemical nanosensors	Nutraceutical nanoencapsulation for enhancement of absorption and stability	[83,281,282,283]
Crop growth and soil condition monitoring using nanosensors	Replacement of meat cholesterol by plant-based steroid containing nanocapsules	Surface coated nanoparticles for antifungal and antimicrobial effect	Coiled nanoparticles (nano-cochleate) for cellular delivery of nutrients	[83,265,284,285]
Nanosensors for detection of plant and animal pathogens Vaccine delivery using nanocapsules	Removal of pathogens by selective binding of nanoparticles from food	Heat resistant films with silicate nanoparticles	Improvement of absorption by dispersing vitamin sprays to nanodroplets	[286,287,288,289]

**Table 8:** Nanoparticles, their toxicity mechanisms and applications.

Nanoparticle Type	Toxicity Mechanism	Applications	Refs
Aluminum oxide nanoparticles	Genotoxicity, changes in protein expression, oxidative stress, cell viability, mitochondrial function	Polymers, biomaterials, fuel cells, paints, textiles, and coatings	[290,291,292,293]
Gold nanoparticles	Non-toxic spherical core, relatively safe; lipid peroxidation, autophagy in lung fibroblasts	Contrast agents and drug carriers	[294,295]
Copper oxide nanoparticles	Oxidative damage (stress), cytotoxicity (cell membrane integrity), nephrotoxicity, genotoxicity, hepatotoxicity, and spleen toxicity	Antibacterial, semiconductors, heat transfer fluids, and contraceptive devices	[296,297,298,299]
Silver nanoparticles	Oxidative stress, genotoxicity, cell viability decrease, nephrotoxicity, cell membrane integrity, lung toxicity, and cardiovascular toxicity	Wound dressing, prostheses, coating for surgical instruments, and antibacterial agents	[300,301,302,303]
Zinc oxide nanoparticles	Mitochondrial dysfunction, genotoxicity, oxidative stress, hepatotoxicity, cell membrane integrity, cell viability, cardiovascular toxicity, inflammation, neurotoxicity, cytotoxicity, and reactive oxygen species production	Sunscreens, gas filters, UV detectors, wave filters, and body care products	[303,304,305,306]
Iron oxide nanoparticles	Neurotoxicity, mitochondrial function alterations, genotoxicity, lung toxicity, hepatotoxicity, reactive oxygen species production, cell viability, and endothelial permeability	Diagnostic agents and drug carriers	[307,308,309]
Titanium nanoparticles	Reactive oxygen species production, nephrotoxicity, genotoxicity, hepatotoxicity, immune function changes, lung toxicity, spleen toxicity, and cardiovascular toxicity	Coloring and pigment agents	[303,304]
Carbon-based nanoparticles and fullerenes	Cell membrane integrity, cell viability, bone toxicity, genotoxicity, hepatotoxicity, nephrotoxicity, spleen toxicity, cardiotoxicity, epigenetic toxicity, skin toxicity, carcinogenesis, neurotoxicity, and immunotoxicity	Drug carriers	[310,311,312,313]
Polymeric nanoparticles	Non-toxic, relatively safe, non-inflammatory, non-immunologic, and least toxic	Drug carriers	[314,315]
Nickel oxide nanoparticles	Apoptosis and lipid peroxidation increase	Antibacterial, antifungal, and cytotoxic	[316,317,318,319,320]
Cerium oxide nanoparticles	Apoptosis, cell membrane damage, p38-NRF2 signaling, and inflammation	Antimicrobial, corrosion protection, polishing, and solar cells	[321,322,323,324]

## Data Availability

The data presented in this study are available on request from the corresponding author.

## References

[1] Gaur M., Misra C., Yadav A.B., Swaroop S., Maolmhuaidh F., Bechelany M., Barhoum A. (2021). Biomedical Applications of Carbon Nanomaterials: Fullerenes, Quantum Dots, Nanotubes, Nanofibers, and Graphene. Materials.

[2] Barhoum A., Pal K., Rahier H., Uludag H., Kim I.S., Bechelany M. (2019). Nanofibers as new-generation materials: From spinning and nano-spinning fabrication techniques to emerging applications. Appl. Mater. Today.

[3] Jeevanandam J., Barhoum A., Chan Y.S., Dufresne A., Danquah M.K. (2018). Review on nanoparticles and nanostructured materials: History, sources, toxicity and regulations. Beilstein J. Nanotechnol..

[4] Barhoum A., El-Maghrabi H.H., Nada A.A., Sayegh S., Roualdes S., Renard A., Iatsunskyi I., Coy E., Bechelany M. (2021). Simultaneous hydrogen and oxygen evolution reactions using free-standing nitrogen-doped-carbon–Co/CoOx nanofiber electrodes decorated with palladium nanoparticles. J. Mater. Chem. A.

[5] Prasad S., Kumar V., Kirubanandam S., Barhoum A. (2018). Engineered nanomaterials: Nanofabrication and surface functionalization. Emerging Applications of Nanoparticles and Architecture Nanostructures: Current Prospects and Future Trends.

[6] Cremers V., Rampelberg G., Barhoum A., Walters P., Claes N., de Oliveira T.M., Van Assche G., Bals S., Dendooven J., Detavernier C. (2018). Oxidation barrier of Cu and Fe powder by Atomic Layer Deposition. Surf. Coat. Technol..

[7] Hammani S., Moulai-Mostefa N., Samyn P., Bechelany M., Dufresne A., Barhoum A. (2020). Morphology, Rheology and Crystallization in Relation to the Viscosity Ratio of Polystyrene/Polypropylene Polymer Blends. Materials.

[8] Barhoum A., Van Lokeren L., Rahier H., Dufresne A., Van Assche G. (2015). Roles of in situ surface modification in controlling the growth and crystallization of CaCO_3_ nanoparticles, and their dispersion in polymeric materials. J. Mater. Sci..

[9] Rehan M., Barhoum A., Khattab T., Gätjen L., Wilken R. (2019). Colored, photocatalytic, antimicrobial and UV-protected viscose fibers decorated with Ag/Ag_2_CO_3_ and Ag/Ag_3_PO_4_ nanoparticles. Cellulose.

[10] Abdel-Haleem F.M., Salah A., Rizk M.S., Moustafa H., Bechelany M., Barhoum A. (2019). Carbon-based Nanosensors for Salicylate Determination in Pharmaceutical Preparations. Electroanalysis.

[11] Abdel-Haleem F., Mahmoud S., Abdel-Ghani N., El Nashar R., Bechelany M., Barhoum A. (2021). Polyvinyl Chloride Modified Carbon Paste Electrodes for Sensitive Determination of Levofloxacin Drug in Serum, Urine, and Pharmaceutical Formulations. Sensors.

[12] Abdel-Haleem F.M., Gamal E., Rizk M.S., Madbouly A., El Nashar R.M., Anis B., Elnabawy H.M., Khalil A.S.G., Barhoum A. (2021). Molecularly Imprinted Electrochemical Sensor-Based Fe_2_O_3_@MWCNTs for Ivabradine Drug Determination in Pharmaceutical Formulation, Serum, and Urine Samples. Front. Bioeng. Biotechnol..

[13] (2016). Parikha Mehrotra, Biosensors and their applications—A review. J. Oral Biol. Craniofac. Res..

[14] Rasouli R., Barhoum A., Uludag H. (2018). A review of nanostructured surfaces and materials for dental implants: Surface coating, patterning and functionalization for improved performance. Biomater. Sci..

[15] Rasouli R., Barhoum A., Bechelany M., Dufresne A. (2018). Nanofibers for Biomedical and Healthcare Applications. Macromol. Biosci..

[16] Singh K.R., Nayak V., Singh J., Singh A.K., Singh R.P. (2021). Potentialities of bioinspired metal and metal oxide nanoparticles in biomedical sciences. RSC Adv..

[17] Tan K.X., Barhoum A., Pan S., Danquah M.K. (2018). Risks and toxicity of nanoparticles and nanostructured materials. Emerging Applications of Nanoparticles and Architecture Nanostructures: Current Prospects and Future Trends.

[18] Kim D., Kim J., Park Y.I., Lee N., Hyeon T. (2018). Recent Development of Inorganic Nanoparticles for Biomedical Imaging. ACS Central Sci..

[19] Mihai M.M., Dima M.B., Dima B., Holban A.M. (2019). Nanomaterials for Wound Healing and Infection Control. Materials.

[20] Said M.M., Rehan M., El-Sheikh S.M., Zahran M.K., Abdel-Aziz M.S., Bechelany M., Barhoum A. (2021). Multifunctional Hydroxyapatite/Silver Nanoparticles/Cotton Gauze for Antimicrobial and Biomedical Applications. Nanomaterials.

[21] Kumar S., Bhushan P., Bhattacharya S. (2017). Fabrication of Nanostructures with Bottom-up Approach and Their Utility in Diagnostics, Therapeutics, and Others. Environmental, Chemical and Medical Sensors.

[22] Sawy A.M., Barhoumbb A., Gaber S.A.A., El-Hallouty S.M., Shousha W.G., Maarouf A.A., Khalilaf S.G.A. (2021). Insights of doxorubicin loaded graphene quantum dots: Synthesis, DFT drug interactions, and cytotoxicity. Mater. Sci. Eng. C.

[23] Barhoum A., Van Assche G., Rahier H., Fleisch M., Bals S., Delplancked M.-P., Leroux F., Bahnemann D. (2017). Sol-gel hot injection synthesis of ZnO nanoparticles into a porous silica matrix and reaction mechanism. Mater. Des..

[24] Barhoum A., Melcher J., Van Assche G., Rahier H., Bechelany M., Fleisch M., Bahnemann D. (2016). Synthesis, growth mechanism, and photocatalytic activity of Zinc oxide nanostructures: Porous microparticles versus nonporous nanoparticles. J. Mater. Sci..

[25] Hong G., Antaris A.L., Dai H. (2017). Near-infrared fluorophores for biomedical imaging. Nat. Biomed. Eng..

[26] Malik N., Arfin T., Khan A.U., Grumezescu T. (2019). Graphene nanomaterials: Chemistry and pharmaceutical perspectives. Nanomaterials for Drug Delivery and Therapy.

[27] Yang Y., Wang L., Wan B., Gu Y., Li X. (2019). Optically Active Nanomaterials for Bioimaging and Targeted Therapy. Front. Bioeng. Biotechnol..

[28] Su S., Kang P.M. (2020). Systemic Review of Biodegradable Nanomaterials in Nanomedicine. Nanomaterials.

[29] Siafaka P.I., Okur N.Ü., Karantas I.D., Okur M.E., Gündoğdu E.A. (2021). Current update on nanoplatforms as therapeutic and diagnostic tools: A review for the materials used as nanotheranostics and imaging modalities. Asian J. Pharm. Health Sci..

[30] Yoon H.Y., Jeon S., You D.G., Park J.H., Kwon I.C., Koo H., Kim K. (2017). Inorganic Nanoparticles for Image-Guided Therapy. Bioconjug. Chem..

[31] Snipstad S., Hak S., Baghirov H., Sulheim E., Mørch Y., Lélu S., Von Haartman E., Bäck M., Nilsson K.P.R., Klymchenko A.S. (2016). Labeling nanoparticles: Dye leakage and altered cellular uptake. Cytom. Part A.

[32] Shandilya P., Sambyal S., Sharma R., Mandyal P., Fang B. (2022). Properties, optimized morphologies, and advanced strategies for photocatalytic applications of WO_3_ based photocatalysts. J. Hazard. Mater..

[33] Rees P., Wills J.W., Brown R., Barnes C.M., Summers H.D. (2019). The origin of heterogeneous nanoparticle uptake by cells. Nat. Commun..

[34] Sukhanova A., Bozrova S., Sokolov P., Berestovoy M., Karaulov A., Nabiev I. (2018). Dependence of Nanoparticle Toxicity on Their Physical and Chemical Properties. Nanoscale Res. Lett..

[35] Forest V., Pourchez J. (2017). Preferential binding of positive nanoparticles on cell membranes is due to electrostatic interactions: A too simplistic explanation that does not take into account the nanoparticle protein corona. Mater. Sci. Eng. C.

[36] Foroozandeh P., Aziz A.A. (2018). Insight into Cellular Uptake and Intracellular Trafficking of Nanoparticles. Nanoscale Res. Lett..

[37] Friedman A.D., Claypool S.E., Liu R. (2013). The Smart Targeting of Nanoparticles. Curr. Pharm. Des..

[38] Yoo J., Park C., Yi G., Lee D., Koo H. (2019). Active Targeting Strategies Using Biological Ligands for Nanoparticle Drug Delivery Systems. Cancers.

[39] Spicer C.D., Jumeaux C., Gupta B., Stevens M.M. (2018). Peptide and protein nanoparticle conjugates: Versatile platforms for biomedical applications. Chem. Soc. Rev..

[40] Kher G., Trehan S., Misra A. (2011). Antisense Oligonucleotides and RNA Interference. Challenges in Delivery of Therapeutic Genomics and Proteomics.

[41] Cremers G.A.O., Rosier B.J.H.M., Brillas R.R., Albertazzi L., de Greef T.F.A. (2019). Efficient Small-Scale Conjugation of DNA to Primary Antibodies for Multiplexed Cellular Targeting. Bioconjug. Chem..

[42] Gao J., Yao X., Chen Y., Gao Z., Zhang J. (2020). Near-Infrared Light-Induced Self-Powered Aptasensing Platform for Aflatoxin B1 Based on Upconversion Nanoparticles-Doped Bi_2_S_3_ Nanorods. Anal. Chem..

[43] Yu Z., Eich C., Cruz L.J. (2020). Recent Advances in Rare-Earth-Doped Nanoparticles for NIR-II Imaging and Cancer Theranostics. Front. Chem..

[44] Chinnathambi S., Shirahata N. (2019). Recent advances on fluorescent biomarkers of near-infrared quantum dots for in vitro and in vivo imaging. Sci. Technol. Adv. Mater..

[45] Arvizo R., Bhattacharya R., Mukherjee P. (2010). Gold nanoparticles: Opportunities and challenges in nanomedicine. Expert Opin. Drug Deliv..

[46] Dong H., Sun L.-D., Yan C.-H. (2021). Lanthanide-Doped Upconversion Nanoparticles for Super-Resolution Microscopy. Front. Chem..

[47] El-Sheikh S.M., Barhoum A., El-Sherbiny S., Morsy F., El-Midany A.A.-H., Rahier H. (2019). Preparation of superhydrophobic nanocalcite crystals using Box–Behnken design. Arab. J. Chem..

[48] Rehan M., Khattab T.A., Barohum A., Gätjen L., Wilken R. (2018). Development of Ag/AgX (X = Cl, I) nanoparticles toward antimicrobial, UV-protected and self-cleanable viscose fibers. Carbohydr. Polym..

[49] Wahajuddin, Arora S. (2012). Superparamagnetic iron oxide nanoparticles: Magnetic nanoplatforms as drug carriers. Int. J. Nanomed..

[50] Lim W.Q., Phua S.Z.F., Xu H.V., Sreejith S., Zhao Y. (2015). Recent advances in multifunctional silica-based hybrid nanocarriers for bioimaging and cancer therapy. Nanoscale.

[51] Liang R., Wei M., Evans D.G., Duan X. (2014). Inorganic nanomaterials for bioimaging, targeted drug delivery and therapeutics. Chem. Commun..

[52] Bhunia S.K., Saha A., Maity A., Ray S.C., Jana N.R. (2013). Carbon Nanoparticle-based Fluorescent Bioimaging Probes. Sci. Rep..

[53] Karatutlu A., Barhoum A., Sapelkin A. (2018). Theories of nanoparticle and nanostructure formation in liquid phase. Emerging Applications of Nanoparticles and Architecture Nanostructures: Current Prospects and Future Trends.

[54] Barhoum A., García-Betancourt M.L. (2018). Physicochemical characterization of nanomaterials: Size, morphology, optical, magnetic, and electrical properties. Emerging Applications of Nanoparticles and Architecture Nanostructures: Current Prospects and Future Trends.

[55] Karatutlu A., Barhoum A., Sapelkin A. (2018). Liquid-phase synthesis of nanoparticles and nanostructured materials. Emerging Applications of Nanoparticles and Architecture Nanostructures: Current Prospects and Future Trends.

[56] Tian P., Tang L., Teng K., Lau S. (2018). Graphene quantum dots from chemistry to applications. Mater. Today Chem..

[57] Singh I., Arora R., Dhiman H., Pahwa R. (2018). Carbon Quantum Dots: Synthesis, Characterization and Biomedical Applications. Turk. J. Pharm. Sci..

[58] Jhonsi M.A. (2018). Carbon Quantum Dots for Bioimaging. State of the Art in Nano-Bioimaging.

[59] Ravichandiran P., Subramaniyan S.A., Bella A.P., Johnson P.M., Kim A.R., Shim K.S., Yoo D.J. (2019). Simple Fluorescence Turn-On Chemosensor for Selective Detection of Ba^2+^ Ion and Its Live Cell Imaging. Anal. Chem..

[60] Hubbs A.F., Sargent L.M., Porter D.W., Sager T.M., Chen B.T., Frazer D.G., Castranova V., Sriram K., Nurkiewicz T.R., Reynolds S.H. (2013). Nanotechnology: Toxicologic Pathology. Toxicol. Pathol..

[61] Maldiney T., Richard C., Seguin J., Wattier N., Bessodes M., Scherman D. (2011). Effect of Core Diameter, Surface Coating, and PEG Chain Length on the Biodistribution of Persistent Luminescence Nanoparticles in Mice. ACS Nano.

[62] Heeger A.J. (2001). Semiconducting and Metallic Polymers: The Fourth Generation of Polymeric Materials (Nobel Lecture). Angew. Chemie Int. Ed..

[63] Thomas S.W., Joly G.D., Swager T.M. (2007). Chemical Sensors Based on Amplifying Fluorescent Conjugated Polymers. Chem. Rev..

[64] Feng X., Liu L., Wang S., Zhu D. (2010). Water-soluble fluorescent conjugated polymers and their interactions with biomacromolecules for sensitive biosensors. Chem. Soc. Rev..

[65] Khanbeigi R.A., Abelha T.F., Woods A., Rastoin O., Harvey R.D., Jones M.-C., Forbes B., Green M.A., Collins H., Dailey L.A. (2015). Surface Chemistry of Photoluminescent F8BT Conjugated Polymer Nanoparticles Determines Protein Corona Formation and Internalization by Phagocytic Cells. Biomacromolecules.

[66] Tuncel D., Demir H.V. (2010). Conjugated polymer nanoparticles. Nanoscale.

[67] Du T., Zhao C., Lai L., Luo S., Selke M., Rehman F.U., Li X., Sun Y., Jiang H., Wang X. (2017). Rapid and multimodal in vivo bioimaging of cancer cells through in situ biosynthesis of Zn&Fe nanoclusters. Nano Res..

[68] Feng L., Liu L., Lv F., Bazan G.C., Wang S. (2014). Preparation and Biofunctionalization of Multicolor Conjugated Polymer Nanoparticles for Imaging and Detection of Tumor Cells. Adv. Mater..

[69] Ravichandiran P., Prabakaran D., Maroli N., Kim A.R., Park B.-H., Han M.-K., Ramesh T., Ponpandian S., Yoo D.J. (2021). Mitochondria-targeted acridine-based dual-channel fluorescence chemosensor for detection of Sn^4+^ and Cr_2_O_72_-ions in water and its application in discriminative detection of cancer cells. J. Hazard. Mater..

[70] Rhim W.-K., Kim M., Hartman K.L., Kang K.W., Nam J.-M. (2015). Radionuclide-labeled nanostructures for In Vivo imaging of cancer. Nano Converg..

[71] Xu Y., Wang C., Jiang T., Ran G., Song Q. (2021). Cadmium induced aggregation of orange–red emissive carbon dots with enhanced fluorescence for intracellular imaging. J. Hazard. Mater..

[72] Zhao W., Yu X., Peng S., Luo Y., Li J., Lu L. (2021). Construction of nanomaterials as contrast agents or probes for glioma imaging. J. Nanobiotechnol..

[73] Pratiwi F., Kuo C.W., Chen B.-C., Chen P. (2019). Recent advances in the use of fluorescent nanoparticles for bioimaging. Nanomedicine.

[74] Chen S., Wang H., Hong Y., Tang B.Z. (2016). Fabrication of fluorescent nanoparticles based on AIE luminogens (AIE dots) and their applications in bioimaging. Mater. Horizons.

[75] Caponetti V., Trzcinski J.W., Cantelli A., Tavano R., Papini E., Mancin F., Montalti M. (2019). Self-Assembled Biocompatible Fluorescent Nanoparticles for Bioimaging. Front. Chem..

[76] Lin J., Chen X., Huang P. (2016). Graphene-based nanomaterials for bioimaging. Adv. Drug Deliv. Rev..

[77] Yadav V., Roy S., Singh P., Khan Z., Jaiswal A. (2018). 2D MoS_2_-Based Nanomaterials for Therapeutic, Bioimaging, and Biosensing Applications. Small.

[78] Zhao W., Li A., Zhang A., Zheng Y., Liu J. (2018). Recent Advances in Functional-Polymer-Decorated Transition-Metal Nanomaterials for Bioimaging and Cancer Therapy. ChemMedChem.

[79] Yi Z., Luo Z., Qin X., Chen Q., Liu X. (2020). Lanthanide-Activated Nanoparticles: A Toolbox for Bioimaging, Therapeutics, and Neuromodulation. Accounts Chem. Res..

[80] Xu Y., Li P., Cheng D., Wu C., Lu Q., Yang W., Zhu X., Yin P., Liu M., Li H. (2020). Group IV nanodots: Synthesis, surface engineering and application in bioimaging and biotherapy. J. Mater. Chem. B.

[81] Esmaeili Y., Bidram E., Zarrabi A., Amini A., Cheng C. (2020). Graphene oxide and its derivatives as promising in-vitro bio-imaging platforms. Sci. Rep..

[82] Das A.K., Gavel P.K. (2020). Low molecular weight self-assembling peptide-based materials for cell culture, antimicrobial, anti-inflammatory, wound healing, anticancer, drug delivery, bioimaging and 3D bioprinting applications. Soft Matter.

[83] Tan P., Li H., Wang J., Gopinath S.C. (2021). Silver nanoparticle in biosensor and bioimaging: Clinical perspectives. Biotechnol. Appl. Biochem..

[84] Bao C., Beziere N., del Pino P., Pelaz B., Estrada G., Tian F., Ntziachristos V., de la Fuente J.M., Cui D. (2012). Gold Nanoprisms as Optoacoustic Signal Nanoamplifiers for In Vivo Bioimaging of Gastrointestinal Cancers. Small.

[85] Chen N.-T., Tang K.-C., Chung M.-F., Cheng S.-H., Huang C.-M., Chu C.-H., Chou P.-T., Souris J.S., Chen C.-T., Mou C.-Y. (2014). Enhanced Plasmonic Resonance Energy Transfer in Mesoporous Silica-Encased Gold Nanorod for Two-Photon-Activated Photodynamic Therapy. Theranostics.

[86] Yadav A., Rao C., Verma N.C., Mishra P.M., Nandi C.K. (2020). Magnetofluorescent Nanoprobe for Multimodal and Multicolor Bioimaging. Mol. Imaging.

[87] Klymchenko A.S., Liu F., Collot M., Anton N. (2020). Dye-Loaded Nanoemulsions: Biomimetic Fluorescent Nanocarriers for Bioimaging and Nanomedicine. Adv. Health Mater..

[88] Xu Y.-M., Tan H.W., Zheng W., Liang Z.-L., Yu F.-Y., Wu D.-D., Yao Y., Zhong Q.-H., Yan R., Lau A.T.Y. (2019). Cadmium telluride quantum dot-exposed human bronchial epithelial cells: A further study of the cellular response by proteomics. Toxicol. Res..

[89] Patra J.K., Das G., Fraceto L.F., Campos E.V.R., del Pilar Rodriguez-Torres M., Acosta-Torres L.S., Diaz-Torres L.A., Grillo R., Swamy M.K., Sharma S. (2018). Nano based drug delivery systems: Recent developments and future prospects. J. Nanobiotechnol..

[90] Yao Y., Zhou Y., Liu L., Xu Y., Chen Q., Wang Y., Wu S., Deng Y., Zhang J., Shao A. (2020). Nanoparticle-Based Drug Delivery in Cancer Therapy and Its Role in Overcoming Drug Resistance. Front. Mol. Biosci..

[91] Kim B., Shin J., Wu J., Omstead D.T., Kiziltepe T., Littlepage L.E., Bilgicer B. (2020). Engineering peptide-targeted liposomal nanoparticles optimized for improved selectivity for HER2-positive breast cancer cells to achieve enhanced in vivo efficacy. J. Control. Release.

[92] Deng Y., Zhang X., Shen H., He Q., Wu Z., Liao W., Yuan M. (2020). Application of the Nano-Drug Delivery System in Treatment of Cardiovascular Diseases. Front. Bioeng. Biotechnol..

[93] Deng S., Gigliobianco M.R., Censi R., Di Martino P. (2020). Polymeric Nanocapsules as Nanotechnological Alternative for Drug Delivery System: Current Status, Challenges and Opportunities. Nanomaterials.

[94] du Toit L., Pillay V., Choonara Y., Pillay S., Harilall S.-L. (2007). Patenting of Nanopharmaceuticals in Drug Delivery: No Small Issue. Recent Patents Drug Deliv. Formul..

[95] Narducci D. (2007). An Introduction to Nanotechnologies: What’s in it for Us?. Veter-Res. Commun..

[96] Navya P., Kaphle A., Srinivas S., Bhargava S.K., Rotello V.M., Daima H.K. (2019). Current trends and challenges in cancer management and therapy using designer nanomaterials. Nano Converg..

[97] Wen H., Jung H., Li X. (2015). Drug Delivery Approaches in Addressing Clinical Pharmacology-Related Issues: Opportunities and Challenges. AAPS J..

[98] Debnath S.K., Srivastava R. (2021). Drug Delivery with Carbon-Based Nanomaterials as Versatile Nanocarriers: Progress and Prospects. Front. Nanotechnol..

[99] Marcelo G., Kaplan E., Tarazona M.P., Mendicuti F. (2015). Interaction of gold nanoparticles with Doxorubicin mediated by supramolecular chemistry. Colloids Surf. B Biointerfaces.

[100] Zhang Y., Walker J.B., Minic Z., Liu F., Goshgarian H., Mao G. (2016). Transporter protein and drug-conjugated gold nanoparticles capable of bypassing the blood-brain barrier. Sci. Rep..

[101] Muhammad Z., Raza A., Ghafoor S., Naeem A., Naz S.S., Riaz S., Ahmed W., Rana N.F. (2016). PEG capped methotrexate silver nanoparticles for efficient anticancer activity and biocompatibility. Eur. J. Pharm. Sci..

[102] Prabha G., Raj V. (2017). Sodium alginate–polyvinyl alcohol–bovin serum albumin coated Fe_3_O_4_ nanoparticles as anticancer drug delivery vehicle: Doxorubicin loading and in vitro release study and cytotoxicity to HepG2 and L02 cells. Mater. Sci. Eng. C.

[103] Prabha G., Raj V. (2016). Formation and characterization of β-cyclodextrin (β-CD)-polyethyleneglycol (PEG)-polyethyleneimine (PEI) coated Fe_3_O_4_ nanoparticles for loading and releasing 5-Fluorouracil drug. Biomed. Pharmacother..

[104] Luo X., Matranga C., Tan S., Alba N., Cui X.T. (2011). Carbon nanotube nanoreservior for controlled release of anti-inflammatory dexamethasone. Biomaterials.

[105] Bhirde A.A., Patel S., Sousa A.A., Patel V., Molinolo A.A., Ji Y., Leapman R.D., Gutkind J.S., Rusling J.F. (2010). Distribution and clearance of PEG-single-walled carbon nanotube cancer drug delivery vehicles in mice. Nanomedicine.

[106] Ruzycka M., Kowalik P., Kowalczyk A., Bujak P., Nowicka A., Wojewódzka M., Kruszewski M., Grudzinski I. (2021). Quantum dots as targeted doxorubicin drug delivery nanosystems in human lung cancer cells. Cancer Nanotechnol..

[107] Roozbahani M., Kharaziha M., Emadi R. (2017). pH sensitive dexamethasone encapsulated laponite nanoplatelets: Release mechanism and cytotoxicity. Int. J. Pharm..

[108] Gurdag S., Khandare J., Stapels S., Matherly L.H., Kannan R.M. (2006). Activity of Dendrimer−Methotrexate Conjugates on Methotrexate-Sensitive and -Resistant Cell Lines. Bioconjug. Chem..

[109] Cirstoiu-Hapca A., Buchegger F., Lange N., Bossy L., Gurny R., Delie F. (2010). Benefit of anti-HER2-coated paclitaxel-loaded immuno-nanoparticles in the treatment of disseminated ovarian cancer: Therapeutic efficacy and biodistribution in mice. J. Control. Release.

[110] Wilson B., Samanta M.K., Santhi K., Kumar K.S., Ramasamy M., Suresh B. (2010). Chitosan nanoparticles as a new delivery system for the anti-Alzheimer drug tacrine. Nanomed. NanotechnoL. Biol. Med..

[111] Anderson R., Franch A., Castell M., Perez-Cano F.J., Bräuer R., Pohlers D., Gajda M., Siskos A.P., Katsila T., Tamvakopoulos C. (2010). Liposomal encapsulation enhances and prolongs the anti-inflammatory effects of water-soluble dexamethasone phosphate in experimental adjuvant arthritis. Arthritis Res. Ther..

[112] Zalba S., Contreras-Sandoval A., Haeri A., Hagen T.L.T., Navarro-Blasco I., Koning G., Garrido M.J. (2015). Cetuximab-oxaliplatin-liposomes for epidermal growth factor receptor targeted chemotherapy of colorectal cancer. J. Control. Release.

[113] Zhang Z., Wu Y., Kuang G., Liu S., Zhou D., Chen X., Jing X., Huang Y. (2017). Pt(iv) prodrug-backboned micelle and DCA loaded nanofibers for enhanced local cancer treatment. J. Mater. Chem. B.

[114] Liu S., Zhou G., Liu D., Xie Z., Huang Y., Wang X., Wu W., Jing X. (2012). Inhibition of orthotopic secondary hepatic carcinoma in mice by doxorubicin-loaded electrospun polylactide nanofibers. J. Mater. Chem. B.

[115] Adeel M., Duzagac F., Canzonieri V., Rizzolio F. (2020). Self-Therapeutic Nanomaterials for Cancer Therapy: A Review. ACS Appl. Nano Mater..

[116] Sutradhar K.B., Amin L. (2014). Nanotechnology in Cancer Drug Delivery and Selective Targeting. ISRN Nanotechnol..

[117] Senapati S., Mahanta A.K., Kumar S., Maiti P. (2018). Controlled drug delivery vehicles for cancer treatment and their performance. Signal Transduct. Target. Ther..

[118] Farokhzad O.C., Langer R. (2009). Impact of Nanotechnology on Drug Delivery. ACS Nano.

[119] Gong Y.-K., Winnik F.M. (2011). Strategies in biomimetic surface engineering of nanoparticles for biomedical applications. Nanoscale.

[120] Khan I., Saeed K., Khan I. (2019). Nanoparticles: Properties, applications and toxicities. Arab. J. Chem..

[121] Zhu Y., Liao L. (2015). Applications of Nanoparticles for Anticancer Drug Delivery: A Review. J. Nanosci. Nanotechnol..

[122] Wen R., Umeano A.C., Kou Y., Xu J., Farooqi A.A. (2019). Nanoparticle systems for cancer vaccine. Nanomedicine.

[123] James H.P., John R., Alex A., Anoop K. (2014). Smart polymers for the controlled delivery of drugs—A concise overview. Acta Pharm. Sin. B.

[124] Fleige E., Quadir M.A., Haag R. (2012). Stimuli-responsive polymeric nanocarriers for the controlled transport of active compounds: Concepts and applications. Adv. Drug Deliv. Rev..

[125] Cohen B.E., Bangham A.D. (1972). Diffusion of Small Non-Electrolytes across Liposome Membranes. Nature.

[126] Thanou M. (2013). Nanoparticles for Drug and Gene Delivery. Encyclopedia of Biophysics.

[127] Vahed S.Z., Fathi N., Samiei M., Dizaj S.M., Sharifi S. (2018). Targeted cancer drug delivery with aptamer-functionalized polymeric nanoparticles. J. Drug Target..

[128] Gugulothu D., Barhoum A., Afzal S.M., Venkateshwarlu B., Uludag H. (2018). Structural Multifunctional Nanofibers and their Emerging Applications. Handbook of Nanofibers.

[129] Bubakir M.M., Li H., Barhoum A., Yang W. (2019). Advances in Melt Electrospinning Technique. Handbook of Nanofibers.

[130] Sasikala A.R.K., Unnithan A.R., Yun Y.-H., Park C.H., Kim C.S. (2016). An implantable smart magnetic nanofiber device for endoscopic hyperthermia treatment and tumor-triggered controlled drug release. Acta Biomater..

[131] Kim Y.-J., Ebara M., Aoyagi T. (2013). A Smart Hyperthermia Nanofiber with Switchable Drug Release for Inducing Cancer Apoptosis. Adv. Funct. Mater..

[132] Ankegowda V.M., Kollur S.P., Prasad S.K., Pradeep S., Dhramashekara C., Jain A.S., Prasad A., Srinivasa C., Sridhara Setty P., Gopinath S.M. (2020). Phyto-Mediated Synthesis of Silver Nanoparticles Using Terminalia chebula Fruit Extract and Evaluation of Its Cytotoxic and Antimicrobial Potential. Molecules.

[133] Gagliardi A., Cosco D., Udongo B.P., Dini L., Viglietto G., Paolino D. (2020). Design and Characterization of Glyceryl Monooleate-Nanostructures Containing Doxorubicin Hydrochloride. Pharmaceutics.

[134] Faisalina A., Sonvico F., Colombo P., Amirul A., Wahab H., Majid M. (2020). Docetaxel-Loaded Poly(3HB-co-4HB) Biodegradable Nanoparticles: Impact of Copolymer Composition. Nanomaterials.

[135] Nicosia A., Cavallaro G., Costa S., Utzeri M.A., Cuttitta A., Giammona G., Mauro N. (2020). Carbon Nanodots for On Demand Chemophotothermal Therapy Combination to Elicit Necroptosis: Overcoming Apoptosis Resistance in Breast Cancer Cell Lines. Cancers.

[136] Sui J., He M., Yang Y., Ma M., Guo Z., Zhao M., Liang J., Sun Y., Fan Y., Zhang X. (2020). Reversing P-Glycoprotein-Associated Multidrug Resistance of Breast Cancer by Targeted Acid-Cleavable Polysaccharide Nanoparticles with Lapatinib Sensitization. ACS Appl. Mater. Interfaces.

[137] Tieu T., Wojnilowicz M., Huda P., Thurecht K.J., Thissen H., Voelcker N.H., Cifuentes-Rius A. (2020). Nanobody-displaying porous silicon nanoparticles for the co-delivery of siRNA and doxorubicin. Biomater. Sci..

[138] Dorjsuren B., Chaurasiya B., Ye Z., Liu Y., Li W., Wang C., Shi D., Evans C.E., Webster T.J., Shen Y. (2020). Cetuximab-Coated Thermo-Sensitive Liposomes Loaded with Magnetic Nanoparticles and Doxorubicin for Targeted EGFR-Expressing Breast Cancer Combined Therapy. Int. J. Nanomed..

[139] Chu I.-M., Tseng S.-H., Chou M.-Y. (2015). Cetuximab-conjugated iron oxide nanoparticles for cancer imaging and therapy. Int. J. Nanomed..

[140] Fernandes C., Oliveira C., Benfeito S., Soares P., Garrido J., Borges F. (2014). Nanotechnology and antioxidant therapy: An emerging approach for neurodegenerative diseases. Curr. Med. Chem..

[141] Martinelli C., Pucci C., Battaglini M., Marino A., Ciofani G. (2019). Antioxidants and Nanotechnology: Promises and Limits of Potentially Disruptive Approaches in the Treatment of Central Nervous System Diseases. Adv. Health Mater..

[142] Swain S., Sahu P.K., Beg S., Babu S.M. (2016). Nanoparticles for Cancer Targeting: Current and Future Directions. Curr. Drug Deliv..

[143] Vaiserman A., Koliada A., Zayachkivska A., Lushchak O. (2020). Nanodelivery of Natural Antioxidants: An Anti-aging Perspective. Front. Bioeng. Biotechnol..

[144] Shah S.T., Yehya W.A., Saad O., Simarani K., Chowdhury Z., Alhadi A.A., Al-Ani L.A. (2017). Surface Functionalization of Iron Oxide Nanoparticles with Gallic Acid as Potential Antioxidant and Antimicrobial Agents. Nanomaterials.

[145] Verma A.K. (2014). Anti-oxidant activities of biopolymeric nanoparticles: Boon or bane. J. Pharm. Res..

[146] Sharpe E., Andreescu D., Andreescu S. (2011). Artificial Nanoparticle Antioxidants. Oxidative Stress: Diagnostics, Prevention, and Therapy.

[147] Yusof F., Ismail N. (2015). Antioxidants effects of Platinum Nanoparticles: A Potential Alternative Treatment to Lung Diseases. J. Appl. Pharm. Sci..

[148] Watanabe A., Kajita M., Kim J., Kanayama A., Takahashi K., Mashino T., Miyamoto Y. (2009). In vitro free radical scavenging activity of platinum nanoparticles. Nanotechnology.

[149] Patlolla A.K., Hackett D., Tchounwou P.B. (2015). Genotoxicity study of silver nanoparticles in bone marrow cells of Sprague–Dawley rats. Food Chem. Toxicol..

[150] Saikia J.P., Paul S., Konwar B.K., Samdarshi S.K. (2010). Nickel oxide nanoparticles: A novel antioxidant. Colloids Surf. B Biointerfaces.

[151] Keshari A.K., Srivastava R., Singh P., Yadav V.B., Nath G. (2018). Antioxidant and antibacterial activity of silver nanoparticles synthesized by Cestrum nocturnum. J. Ayurveda Integr. Med..

[152] Kumar H., Bhardwaj K., Nepovimova E., Kuca K., Dhanjal D.S., Bhardwaj S., Bhatia S.K., Verma R., Kumar D. (2020). Antioxidant Functionalized Nanoparticles: A Combat against Oxidative Stress. Nanomaterials.

[153] Khan M.S., Vishakante G.D., Siddaramaiah H. (2013). Gold nanoparticles: A paradigm shift in biomedical applications. Adv. Colloid Interface Sci..

[154] Khalil I., Yehye W.A., Etxeberria A.E., Alhadi A.A., Dezfooli S.M., Julkapli N.B.M., Basirun W.J., Seyfoddin A. (2019). Nanoantioxidants: Recent Trends in Antioxidant Delivery Applications. Antioxidants.

[155] Arriagada F., Günther G., Morales J. (2020). Nanoantioxidant–Based Silica Particles as Flavonoid Carrier for Drug Delivery Applications. Pharmaceutics.

[156] Yi X., Zimmerman M.C., Yang R., Tong J., Vinogradov S., Kabanov A.V. (2010). Pluronic-modified superoxide dismutase 1 attenuates angiotensin II-induced increase in intracellular superoxide in neurons. Free. Radic. Biol. Med..

[157] Tong J., Yi X., Luxenhofer R., Banks W.A., Jordan R., Zimmerman M.C., Kabanov A.V. (2012). Conjugates of Superoxide Dismutase 1 with Amphiphilic Poly(2-oxazoline) Block Copolymers for Enhanced Brain Delivery: Synthesis, Characterization and Evaluation in Vitro and in Vivo. Mol. Pharm..

[158] Christofidou-Solomidou M., Scherpereel A., Wiewrodt R., Ng K., Sweitzer T., Arguiri E., Shuvaev V., Solomides C.C., Albelda S.M., Muzykantov V.R. (2003). PECAM-directed delivery of catalase to endothelium protects against pulmonary vascular oxidative stress. Am. J. Physiol. Cell. Mol. Physiol..

[159] Brynskikh A.M., Zhao Y., Mosley R.L., Li S., Boska M.D., Klyachko N.L., Kabanov A.V., Gendelman H.E., Batrakova E.V. (2010). Macrophage delivery of therapeutic nanozymes in a murine model of Parkinson’s disease. Nanomedicine.

[160] Williams S.R., Lepene B.S., Thatcher C.D., Long T.E. (2008). Synthesis and Characterization of Poly(ethylene glycol)−Glutathione Conjugate Self-Assembled Nanoparticles for Antioxidant Delivery. Biomacromolecules.

[161] Gonnet M., Lethuaut L., Boury F. (2010). New trends in encapsulation of liposoluble vitamins. J. Control Release.

[162] Wegmann J., Krucker M., Bachmann S., Fischer G., Zeeb D., Lienau A., Glaser T., Runge F., Lüddecke E., Albert K. (2002). Characterization of Lycopene Nanoparticles Combining Solid-State and Suspended-State NMR Spectroscopy. J. Agric. Food Chem..

[163] Mignet N., Seguin J., Romano M.R., Brullé L., Touil Y., Scherman D., Bessodes M., Chabot G.G. (2012). Development of a liposomal formulation of the natural flavonoid fisetin. Int. J. Pharm..

[164] Deligiannakis Y., Sotiriou G.A., Pratsinis S.E. (2012). Antioxidant and Antiradical SiO_2_ Nanoparticles Covalently Functionalized with Gallic Acid. ACS Appl. Mater. Interfaces.

[165] Liu P., Tang H., Lu M., Gao C., Wang F., Ding Y., Zhang S., Yang M. (2016). Preparation of nanosilica-immobilized antioxidant and the antioxidative behavior in low density polyethylene. Polym. Degrad. Stab..

[166] Du L., Suo S., Wang G., Jia H., Liu K.J., Zhao B., Liu Y. (2013). Mechanism and Cellular Kinetic Studies of the Enhancement of An-tioxidant Activity by Using Surface-Functionalized Gold Nanoparticles. Chem. Eur. J..

[167] Sahiner N., Sagbas S., Aktas N. (2016). Preparation and characterization of monodisperse, mesoporous natural poly (tannic acid)–silica nanoparticle composites with antioxidant properties. Microporous Mesoporous Mater..

[168] Arriagada F., Correa O., Gunther G., Nonell S., Mura F., Olea-Azar C., Morales J. (2016). Morin Flavonoid Adsorbed on Mesoporous Silica, a Novel Antioxidant Nanomaterial. PLoS ONE.

[169] Kang D.-W., Kim C.K., Jeong H.-G., Soh M., Kim T., Choi I.-Y., Ki S.-K., Kim D.Y., Yang W., Hyeon T. (2017). Biocompatible custom ceria nanoparticles against reactive oxygen species resolve acute inflammatory reaction after intracerebral hemorrhage. Nano Res..

[170] Joseph A., Wood T., Chen C.-C., Corry K., Snyder J.M., Juul S.E., Parikh P., Nance E. (2018). Curcumin-loaded polymeric nanoparticles for neuroprotection in neonatal rats with hypoxic-ischemic encephalopathy. Nano Res..

[171] Yalcinkaya F., Komarek M., Lubasová D., Sanetrnik F., Maryska J. (2016). Preparation of Antibacterial Nanofibre/Nanoparticle Covered Composite Yarns. J. Nanomater..

[172] Salama A., Abouzeid R., Leong W.S., Jeevanandam J., Samyn P., Dufresne A., Bechelany M., Barhoum A. (2021). Nanocellulose-Based Materials for Water Treatment: Adsorption, Photocatalytic Degradation, Disinfection, Antifouling, and Nanofiltration. Nanomaterials.

[173] Shahriary M., Veisi H., Hekmati M., Hemmati S. (2018). In situ green synthesis of Ag nanoparticles on herbal tea extract (Stachys lavandulifolia)-modified magnetic iron oxide nanoparticles as antibacterial agent and their 4-nitrophenol catalytic reduction activity. Mater. Sci. Eng. C.

[174] Youssef A.M., Moustafa H.A., Barhoum A., Hakim A.E.-F.A.A., Dufresne A. (2017). Evaluation of the Morphological, Electrical and Antibacterial Properties of Polyaniline Nanocomposite Based on Zn/Al-Layered Double Hydroxides. ChemistrySelect.

[175] Sudha P.N., Sangeetha K., Vijayalakshmi K., Barhoum A. (2018). Nanomaterials History, Classification, Unique Properties, Production and Market. Emerging Applications of Nanoparticles and Architecture Nanostructures: Current Prospects and Future Trends.

[176] Nnaji C.O., Jeevanandam J., Chan Y.S., Danquah M.K., Pan S., Barhoum A. (2018). Engineered nanomaterials for wastewater treatment: Current and future trends. Fundamentals of Nanoparticles.

[177] Jung W.K., Koo H.C., Kim K.W., Shin S., Kim S.H., Park Y.H. (2008). Antibacterial Activity and Mechanism of Action of the Silver Ion in *Staphylococcus aureus* and *Escherichia coli*. Appl. Environ. Microbiol..

[178] Smetana A.B., Klabunde K.J., Marchin G.R., Sorensen C.M. (2008). Biocidal Activity of Nanocrystalline Silver Powders and Particles. Langmuir.

[179] Panacek A., Kvitek L., Prucek R., Kolar M., Vecerova R., Pizurova N., Sharma V.K., Nevecna T., Zboril R. (2006). Silver colloid nanoparticles: Synthesis, characterization, and their antibacterial activity. J. Phys. Chem. B.

[180] Nanda A., Saravanan M. (2009). Biosynthesis of silver nanoparticles from Staphylococcus aureus and its antimicrobial activity against MRSA and MRSE. Nanomed. Nanotechnol. Biol. Med..

[181] Matsunaga T., Tomoda R., Nakajima T., Nakamura N., Komine T. (1988). Continuous-sterilization system that uses photosemiconductor powders. Appl. Environ. Microbiol..

[182] Kim B., Kim D., Cho D., Cho S. (2003). Bactericidal effect of TiO_2_ photocatalyst on selected food-borne pathogenic bacteria. Chemosphere.

[183] Sunada K., Kikuchi Y., Hashimoto K., Fujishima A. (1998). Bactericidal and Detoxification Effects of TiO_2_ Thin Film Photocatalysts. Environ. Sci. Technol..

[184] Ren G., Hu D., Cheng E.W., Vargas-Reus M.A., Reip P., Allaker R.P. (2009). Characterisation of copper oxide nanoparticles for antimicrobial applications. Int. J. Antimicrob. Agents.

[185] Cioffi N., Torsi L., Ditaranto N., Tantillo G., Ghibelli L., Sabbatini L., Bleve-Zacheo T., D’Alessio M., Zambonin A.P.G., Traversa E. (2005). Copper Nanoparticle/Polymer Composites with Antifungal and Bacteriostatic Properties. Chem. Mater..

[186] Rajakumar G., Rahuman A.A., Roopan S.M., Khanna V.G., Elango G., Kamaraj C., Zahir A.A., Velayutham K. (2012). Fungus-mediated biosynthesis and characterization of TiO_2_ nanoparticles and their activity against pathogenic bacteria. Spectrochim. Acta Part A Mol. Biomol. Spectrosc..

[187] Gómez-Ortíz N., De la Rosa-García S., González-Gómez W., Soria-Castro M., Quintana P., Oskam G., Ortega-Morales B. (2013). Antifungal Coatings Based on Ca(OH)_2_ Mixed with ZnO/TiO_2_ Nanomaterials for Protection of Limestone Monuments. ACS Appl. Mater. Interfaces.

[188] Herman A., Herman A.P. (2014). Nanoparticles as Antimicrobial Agents: Their Toxicity and Mechanisms of Action. J. Nanosci. Nanotechnol..

[189] Mageshwari K., Sathyamoorthy R. (2013). Flower-shaped CuO Nanostructures: Synthesis, Characterization and Antimicrobial Activity. J. Mater. Sci. Technol..

[190] Panáček A., Kolář M., Večeřová R., Prucek R., Soukupová J., Kryštof V., Hamal P., Zbořil R., Kvítek L. (2009). Antifungal activity of silver nanoparticles against *Candida* spp.. Biomaterials.

[191] Sawai J., Yoshikawa T. (2004). Quantitative evaluation of antifungal activity of metallic oxide powders (MgO, CaO and ZnO) by an indirect conductimetric assay. J. Appl. Microbiol..

[192] Sawai J., Yoshikawa T. (2003). Measurement of fungi by an indirect conductimetric assay. Lett. Appl. Microbiol..

[193] Di Gianvincenzo P., Marradi M., Martínez-Ávila O.M., Bedoya L.M., Alcami J., Penadés S. (2010). Gold nanoparticles capped with sulfate-ended ligands as anti-HIV agents. Bioorg. Med. Chem. Lett..

[194] Lara H.H., Ayala-Nuñez N.V., Ixtepan-Turrent L., Rodriguez-Padilla C. (2010). Mode of antiviral action of silver nanoparticles against HIV-1. J. Nanobiotechnol..

[195] Lu L., Sun R.W.-Y., Chen R., Hui C.-K., Ho C.-M., Luk J.M., Lau G.K.K., Che C.-M. (2008). Silver nanoparticles inhibit hepatitis B virus replication. Antivir. Ther..

[196] Zheng Y., Cloutier P., Hunting D.J., Sanche L. (2008). Radiosensitization by Gold Nanoparticles: Comparison of DNA Damage Induced by Low and High-Energy Electrons. J. Biomed. Nanotechnol..

[197] Syngouna V.I., Chrysikopoulos C.V. (2017). Inactivation of MS2 bacteriophage by titanium dioxide nanoparticles in the presence of quartz sand with and without ambient light. J. Colloid Interface Sci..

[198] Cui H., Jiang J., Gu W., Sun C., Wu D., Yang T., Yang G. (2010). Photocatalytic Inactivation Efficiency of Anatase Nano-TiO_2_ Sol on the H9N2 Avian Influenza Virus. Photochem. Photobiol..

[199] Baiocco P., Ilari A., Ceci P., Orsini S., Gramiccia M., Di Muccio T., Colotti G. (2010). Inhibitory Effect of Silver Nanoparticles on Trypanothione Reductase Activity and Leishmania infantum Proliferation. ACS Med. Chem. Lett..

[200] Allahverdiyev A.M., Abamor E., Bagirova M., Ustundag C.B., Kaya C., Rafailovich M. (2011). Antileishmanial effect of silver nanoparticles and their enhanced antiparasitic activity under ultraviolet light. Int. J. Nanomed..

[201] Nadhman A., Nazir S., Khan M.I., Arooj S., Bakhtiar M., Shahnaz G., Yasinzai M. (2014). PEGylated silver doped zinc oxide nanoparticles as novel photosensitizers for photodynamic therapy against Leishmania. Free. Radic. Biol. Med..

[202] Saad A.H.A., Soliman M.I., Azzam A.M. (2015). Antiparasitic Activity of Silver and Copper Oxide Nanoparticles against Entamoeba histolytica and Cryptosporidium parvum Cysts. J. Egypt. Soc. Parasitol..

[203] Ramyadevi J., Jeyasubramanian K., Marikani A., Rajakumar G., Rahuman A.A., Santhoshkumar T., Kirthi A.V., Jayaseelan C., Marimuthu S. (2011). Copper nanoparticles synthesized by polyol process used to control hematophagous parasites. Parasitol. Res..

[204] Barhoum A., Rehan M.F., Rahier H., Bechelany M., Van Assche G. (2016). Seed-Mediated Hot-Injection Synthesis of Tiny Ag Nanocrystals on Nanoscale Solid Supports and Reaction Mechanism. ACS Appl. Mater. Interfaces.

[205] Rehan M., Barhoum A., Van Assche G., Dufresne A., Gätjen L., Wilken R. (2017). Towards multifunctional cellulosic fabric: UV photo-reduction and in-situ synthesis of silver nanoparticles into cellulose fabrics. Int. J. Biol. Macromol..

[206] Barhoum A., Rahier H., Benelmekki M., Van Assche G. (2018). Recent trends in nanostructured particles: Synthesis, functionalization, and applications. Fundamentals of Nanoparticles.

[207] Rastogi A., Singh P., Haraz F.A., Barhoum A. Chapter 19—Biological Synthesis of Nanoparticles: An Environmentally Benign Approach. https://www.sciencedirect.com/science/article/pii/B9780323512558000239.

[208] Dakal T.C., Kumar A., Majumdar R.S., Yadav V. (2016). Mechanistic Basis of Antimicrobial Actions of Silver Nanoparticles. Front. Microbiol..

[209] Möhler J.S., Sim W., Blaskovich M.A., Cooper M.A., Ziora Z.M. (2018). Silver bullets: A new lustre on an old antimicrobial agent. Biotechnol. Adv..

[210] Gupta N., Upadhyaya C.P., Singh A., Abd-Elsalam K.A., Prasad R. (2018). Applications of Silver Nanoparticles in Plant Protection. Nanobiotechnology Applications in Plant Protection.

[211] Abdel-Aziz M., Shaheen M.S., El-Nekeety A.A., Abdel-Wahhab M.A. (2013). Antioxidant and antibacterial activity of silver nanoparticles biosynthesized using Chenopodium murale leaf extract. J. Saudi Chem. Soc..

[212] Belaiche Y., Khelef A., Laouini S.E., Bouafia A., Tedjani M.L., Barhoum A. (2021). Green synthesis and characterization of silver/silver oxide nanoparticles using aqueous leaves extract of artemisia herbaalba as reducing and capping agents. Rom. J. Mater..

[213] Fatima S., Ali K., Ahmed B., Al Kheraif A.A., Syed A., Elgorban A.M., Musarrat J., Lee J. (2021). Titanium Dioxide Nanoparticles Induce Inhibitory Effects against Planktonic Cells and Biofilms of Human Oral Cavity Isolates of *Rothia mucilaginosa*, *Georgenia* sp. and *Staphylococcus saprophyticus*. Pharmaceutics.

[214] Al-Shabib N.A., Husain F.M., Qais F.A., Ahmad N., Khan A., Alyousef A.A., Arshad M., Noor S., Khan J.M., Alam P. (2020). Phyto-Mediated Synthesis of Porous Titanium Dioxide Nanoparticles From Withania somnifera Root Extract: Broad-Spectrum Attenuation of Biofilm and Cytotoxic Properties Against HepG2 Cell Lines. Front. Microbiol..

[215] Ilyas M., Waris A., Khan A.U., Zamel D., Yar L., Baset A., Muhaymin A., Khan S., Ali A., Ahmad A. (2021). Biological synthesis of titanium dioxide nanoparticles from plants and microorganisms and their potential biomedical applications. Inorg. Chem. Commun..

[216] Balaraman R.P., Mendel J., Flores L., Choudhary M. (2021). Nanoparticle Biosynthesis and Interaction with the Microbial Cell, Antimicrobial and Antibiofilm Effects, and Environmental Impact. Nanomaterial Biointeractions at the Cellular, Organismal and System Levels.

[217] Jardón-Maximino N., Cadenas-Pliego G., Ávila-Orta C., Comparán-Padilla V., Lugo-Uribe L., Pérez-Alvarez M., Tavizón S., Santillán G. (2021). Antimicrobial Property of Polypropylene Composites and Functionalized Copper Nanoparticles. Polymers.

[218] Gharpure S., Akash A., Ankamwar B. (2020). A Review on Antimicrobial Properties of Metal Nanoparticles. J. Nanosci. Nanotechnol..

[219] El-Sheikh S., El-Sherbiny S., Barhoum A., Deng Y. (2013). Effects of cationic surfactant during the precipitation of calcium carbonate nano-particles on their size, morphology, and other characteristics. Colloids Surf. A Physicochem. Eng. Asp..

[220] Sharma U., Badyal P.N., Gupta S. (2015). Polymeric nanoparticles drug delivery to brain: A review. Int. J. Pharmacol..

[221] Jun A.S., Larkin D.F.P. (2003). Prospects for gene therapy in corneal disease. Eye.

[222] Dzau V.J., Mann M.J., Morishita R., Kaneda Y. (1996). Fusigenic viral liposome for gene therapy in cardiovascular diseases. Proc. Natl. Acad. Sci. USA.

[223] Caplen N., Gao X., Hayes P., Elaswarapu R., Fisher G., Kinrade E., Chakera A., Schorr J., Hughes B., Dorin J.R. (1994). Gene therapy for cystic fibrosis in humans by liposome-mediated DNA transfer: The production of resources and the regulatory process. Gene Ther..

[224] Balazs D.A., Godbey W. (2010). Liposomes for Use in Gene Delivery. J. Drug Deliv..

[225] Ito I., Ji L., Tanaka F., Saito Y., Gopalan B., Branch C.D., Xu K., Atkinson E.N., Bekele B.N., Stephens L.C. (2004). Liposomal vector mediated delivery of the 3p FUS1 gene demonstrates potent antitumor activity against human lung cancer in vivo. Cancer Gene Ther..

[226] Singh B.N., Prateeksha, Gupta V.K., Chen J., Atanasov A. (2017). Organic Nanoparticle-Based Combinatory Approaches for Gene Therapy. Trends Biotechnol..

[227] Chen J., Guo Z., Tian H., Chen X. (2016). Production and clinical development of nanoparticles for gene delivery. Mol. Ther. Methods Clin. Dev..

[228] Nagamune T. (2017). Biomolecular engineering for nanobio/bionanotechnology. Nano Converg..

[229] Prabu S.L., Suriyaprakash T.N.K., Thirumurugan R. (2017). Medicated Nanoparticle for Gene Delivery. Advanced Technology for Delivering Therapeutics.

[230] Jat S.K., Bhattacharya J., Sharma M.K. (2020). Nanomaterial based gene delivery: A promising method for plant genome engineering. J. Mater. Chem. B.

[231] Abdel-Haleem F.M., Gamal E., Rizk M.S., El Nashar R.M., Anis B., Elnabawy H.M., Khalil A.S., Barhoum A. (2020). t-Butyl calixarene/Fe_2_O_3_@MWCNTs composite-based potentiometric sensor for determination of ivabradine hydrochloride in pharmaceutical formulations. Mater. Sci. Eng. C.

[232] Abdel-Haleem F.M., Saad M., Barhoum A., Bechelany M., Rizk M.S. (2018). PVC membrane, coated-wire, and carbon-paste ion-selective electrodes for potentiometric determination of galantamine hydrobromide in physiological fluids. Mater. Sci. Eng. C.

[233] El Nashar R.M., Ghani N.T.A., El Gohary N.A., Barhoum A., Madbouly A. (2017). Molecularly imprinted polymers based biomimetic sensors for mosapride citrate detection in biological fluids. Mater. Sci. Eng. C.

[234] El-Beshlawy M.M., Abdel-Haleem F.M., Barhoum A. (2021). Molecularly Imprinted Potentiometric Sensor for Nanomolar Determination of Pioglitazone Hydrochloride in Pharmaceutical Formulations. Electroanalysis.

[235] Naresh V., Lee N. (2021). A Review on Biosensors and Recent Development of Nanostructured Materials-Enabled Biosensors. Sensors.

[236] Abdel-Karim R., Reda Y., Abdel-Fattah A. (2020). Review—Nanostructured Materials-Based Nanosensors. J. Electrochem. Soc..

[237] Tang C.K., Vaze A., Shen M., Rusling J.F. (2016). High-Throughput Electrochemical Microfluidic Immunoarray for Multiplexed Detection of Cancer Biomarker Proteins. ACS Sens..

[238] Wang Z., Hu T., Liang R., Wei M. (2020). Application of Zero-Dimensional Nanomaterials in Biosensing. Front. Chem..

[239] Cotta M.A. (2020). Quantum Dots and Their Applications: What Lies Ahead?. ACS Appl. Nano Mater..

[240] Abraham J., Arunima R., Nimitha K., George S.C., Thomas S. (2021). One-dimensional (1D) nanomaterials: Nanorods and nanowires. Nanoscale Processing.

[241] Erol O., Uyan I., Hatip M., Yilmaz C., Tekinay A.B., Guler M.O. (2017). Recent advances in bioactive 1D and 2D carbon nanomaterials for biomedical applications. Nanomed. Nanotechnol. Biol. Med..

[242] Dvir T., Timko B.P., Kohane D.S., Langer R. (2011). Nanotechnological strategies for engineering complex tissues. Nat. Nanotechnol..

[243] Shi J., Votruba A.R., Farokhzad O., Langer R. (2010). Nanotechnology in Drug Delivery and Tissue Engineering: From Discovery to Applications. Nano Lett..

[244] Zhang K., Barhoum A., Xiaoqing C., Li H., Samyn P. (2019). Cellulose Nanofibers: Fabrication and Surface Functionalization Techniques. Handbook of Nanofibers.

[245] Gugulothu D., Barhoum A., Nerella R., Ajmer R., Bechlany M. (2018). Fabrication of Nanofibers: Electrospinning and Non-Electrospinning Techniques. Handbook of Nanofibers.

[246] Meftahi A., Samyn P., Geravand S.A., Khajavi R., Alibkhshi S., Bechelany M., Barhoum A. (2021). Nanocelluloses as skin biocompatible materials for skincare, cosmetics, and healthcare: Formulations, regulations, and emerging applications. Carbohydr. Polym..

[247] Rabie A.M.I., Ali A.S.M., Al-Zeer M.A., Barhoum A., El-Hallouty S., Shousha W.G., Berg J., Kurreck J., Khalil A.S.G. (2022). Spontaneous Formation of 3D Breast Cancer Tissues on Electrospun Chitosan/Poly(ethylene oxide) Nanofibrous Scaffolds. ACS Omega.

[248] Barhoum A., García-Betancourt M.L., Jeevanandam J., Hussien E.A., Mekkawy S.A., Mostafa M., Omran M.M., Abdalla M.S., Bechelany M. (2022). Review on Natural, Incidental, Bioinspired, and Engineered Nanomaterials: History, Definitions, Classifications, Synthesis, Properties, Market, Toxicities, Risks, and Regulations. Nanomaterials.

[249] Besinis A., De Peralta T., Tredwin C.J., Handy R.D. (2015). Review of Nanomaterials in Dentistry: Interactions with the Oral Microenvironment, Clinical Applications, Hazards, and Benefits. ACS Nano.

[250] Xi Y., Wang Y., Gao J., Xiao Y., Du J. (2019). Dual Corona Vesicles with Intrinsic Antibacterial and Enhanced Antibiotic Delivery Capabilities for Effective Treatment of Biofilm-Induced Periodontitis. ACS Nano.

[251] Lombardi V.R. (2012). Editorial [Exploring Neural-Immune System Interactions]. Curr. Immunol. Rev..

[252] Bertram L. (2005). The genetic epidemiology of neurodegenerative disease. J. Clin. Investig..

[253] Faroni A., Mobasseri S.A., Kingham P.J., Reid A.J. (2015). Peripheral nerve regeneration: Experimental strategies and future perspectives. Adv. Drug Deliv. Rev..

[254] Barhoum A., El-Maghrabi H.H., Iatsunskyi I., Coy E., Renard A., Salameh C., Weber M., Sayegh S., Nada A., Roualdes S. (2020). Atomic layer deposition of Pd nanoparticles on self-supported carbon-Ni/NiO-Pd nanofiber electrodes for electrochemical hydrogen and oxygen evolution reactions. J. Colloid Interface Sci..

[255] Barhoum A., Rasouli R., Yousefzadeh M., Rahier H., Bechelany M. (2019). Nanofiber Technologies: History and Development. Handbook of Nanofibers.

[256] Barhoum A., Favre T., Sayegh S., Tanos F., Coy E., Iatsunskyi I., Razzouk A., Cretin M., Bechelany M. (2021). 3D Self-Supported Nitrogen-Doped Carbon Nanofiber Electrodes Incorporated Co/CoO_x_ Nanoparticles: Application to Dyes Degradation by Electro-Fenton-Based Process. Nanomaterials.

[257] Turky A.O., Barhoum A., Rashad M., Bechelany M. (2017). Enhanced the structure and optical properties for ZnO/PVP nanofibers fabricated via electrospinning technique. J. Mater. Sci. Mater. Electron..

[258] Barhoum A., Jeevanandam J., Rastogi A., Samyn P., Boluk Y., Dufresne A., Danquah M.K., Bechelany M. (2020). Plant celluloses, hemicelluloses, lignins, and volatile oils for the synthesis of nanoparticles and nanostructured materials. Nanoscale.

[259] Aljabali A.A.A., Obeid M.A., Al Zoubi M.S., Charbe N.B., Chellappan D.K., Mishra V., Dureja H., Gupta G., Prasher P., Dua K. (2021). Nanocelluloses in Sensing Technology. Handbook of Nanocelluloses.

[260] Xue J., Pisignano D., Xia Y. (2020). Maneuvering the Migration and Differentiation of Stem Cells with Electrospun Nanofibers. Adv. Sci..

[261] Sorg H., Tilkorn D.J., Hager S., Hauser J., Mirastschijski U. (2016). Skin Wound Healing: An Update on the Current Knowledge and Concepts. Eur. Surg. Res..

[262] Dreifke M.B., Jayasuriya A.A., Jayasuriya A.C. (2014). Current wound healing procedures and potential care. Mater. Sci. Eng. C.

[263] Pober J.S., Sessa W.C. (2014). Inflammation and the blood microvascular system. Cold Spring Harb. Perspect Biol..

[264] Bodnar R.J. (2015). Chemokine Regulation of Angiogenesis during Wound Healing. Adv. Wound Care.

[265] Mechanick J.I. (2004). Practical aspects of nutritional support for wound-healing patients. Am. J. Surg..

[266] Greer N., Foman N.A., Macdonald R., Dorrian J., Fitzgerald P., Rutks I., Wilt T.J. (2013). Advanced Wound Care Therapies for Nonhealing Diabetic, Venous, and Arterial Ulcers. Ann. Intern. Med..

[267] Ravichandiran P., Prabakaran D., Maroli N., Boguszewska-Czubara A., Masłyk M., Kim A.R., Chandrasekaran B., Yoo D.J. (2021). Construction of a simple dual-channel fluorescence chemosensor for Cu^2+^ ion and GSSG detection and its mitochondria-targeting bioimaging applications. Anal. Chim. Acta.

[268] Randeria P.S., Seeger M.A., Wang X.-Q., Wilson H., Shipp D., Mirkin C.A., Paller A.S. (2015). siRNA-based spherical nucleic acids reverse impaired wound healing in diabetic mice by ganglioside GM3 synthase knockdown. Proc. Natl. Acad. Sci. USA.

[269] Wang P., Lombi E., Zhao F.-J., Kopittke P. (2016). Nanotechnology: A New Opportunity in Plant Sciences. Trends Plant Sci..

[270] Rastogi A., Tripathi D.K., Yadav S., Chauhan D.K., Živčák M., Ghorbanpour M., El-Sheery N.I., Brestic M. (2019). Application of silicon nanoparticles in agriculture. 3 Biotech.

[271] Jampílek J., Kráľová K. (2017). Nanomaterials for delivery of nutrients and growth-promoting compounds to plants. Nano-Technology.

[272] Liu R., Lal R. (2015). Potentials of engineered nanoparticles as fertilizers for increasing agronomic productions. Sci. Total Environ..

[273] Saharan V., Kumaraswamy R.V., Choudhary R.C., Kumari S., Pal A., Raliya R., Biswas P. (2016). Cu-Chitosan Nanoparticle Mediated Sustainable Approach To Enhance Seedling Growth in Maize by Mobilizing Reserved Food. J. Agric. Food Chem..

[274] Verma S.K., Das A.K., Patel M.K., Shah A., Kumar V., Gantait S. (2018). Engineered nanomaterials for plant growth and development: A perspective analysis. Sci. Total Environ..

[275] Khodakovskaya M.V., Kim B.-S., Kim J.N., Alimohammadi M., Dervishi E., Mustafa T., Cernigla C.E. (2012). Carbon Nanotubes as Plant Growth Regulators: Effects on Tomato Growth, Reproductive System, and Soil Microbial Community. Small.

[276] Abdel-Aziz H., Hasaneen M.N.A., Omer A.M. (2016). Nano chitosan-NPK fertilizer enhances the growth and productivity of wheat plants grown in sandy soil. Span. J. Agric. Res..

[277] Meurer R.A., Kemper S., Knopp S., Eichert T., Jakob F., Goldbach H.E., Schwaneberg U., Pich A. (2017). Biofunctional Microgel-Based Fertilizers for Controlled Foliar Delivery of Nutrients to Plants. Angew. Chem. Int. Ed..

[278] Shang Y., Hasan M.K., Ahammed G.J., Li M., Yin H., Zhou J. (2019). Applications of Nanotechnology in Plant Growth and Crop Protection: A Review. Molecules.

[279] Qureshi A., Singh D., Dwivedi S. (2018). Nano-fertilizers: A Novel Way for Enhancing Nutrient Use Efficiency and Crop Productivity. Int. J. Curr. Microbiol. Appl. Sci..

[280] Preetha P.S., Balakrishnan N. (2017). A Review of Nano Fertilizers and Their Use and Functions in Soil. Int. J. Curr. Microbiol. Appl. Sci..

[281] Solanki P., Bhargava A., Chhipa H., Jain N., Panwar J. (2015). Nano-fertilizers and Their Smart Delivery System. Nanotechnologies in Food and Agriculture.

[282] Subramanian K.S., Manikandan A., Thirunavukkarasu M., Rahale C.S. (2015). Nano-fertilizers for Balanced Crop Nutrition. Nanotechnologies in Food and Agriculture.

[283] Sanzari I., Leone A., Ambrosone A. (2019). Nanotechnology in Plant Science: To Make a Long Story Short. Front. Bioeng. Biotechnol..

[284] Park B. (2013). Nanotechnology and the packaging of food and other fast-moving consumer goods. Trends in Packaging of Food, Beverages and Other Fast-Moving Consumer Goods (FMCG).

[285] Rossi M., Passeri D., Sinibaldi A., Angjellari M., Tamburri E., Sorbo A., Carata E., Dini L. (2017). Nanotechnology for Food Packaging and Food Quality Assessment. Advances in Food and Nutrition Research.

[286] Contado C. (2015). Nanomaterials in consumer products: A challenging analytical problem. Front. Chem..

[287] Mu L., Droujinine I.A., Rajan N.K., Sawtelle S.D., Reed M.A. (2014). Direct, Rapid, and Label-Free Detection of Enzyme–Substrate Interactions in Physiological Buffers Using CMOS-Compatible Nanoribbon Sensors. Nano Lett..

[288] Huang Q., Yu H., Ru Q. (2010). Bioavailability and Delivery of Nutraceuticals Using Nanotechnology. J. Food Sci..

[289] Inbaraj B.S., Chen B. (2015). Nanomaterial-based sensors for detection of foodborne bacterial pathogens and toxins as well as pork adulteration in meat products. J. Food Drug Anal..

[290] Jampilek J., Kos J., Kralova K. (2019). Potential of Nanomaterial Applications in Dietary Supplements and Foods for Special Medical Purposes. Nanomaterials.

[291] Mali S.C., Raj S., Trivedi R. (2020). Nanotechnology a novel approach to enhance crop productivity. Biochem. Biophys. Rep..

[292] Saifullah M., Shishir M.R.I., Ferdowsi R., Tanver Rahman M.R., Van Vuong Q. (2019). Micro and nano encapsulation, retention and controlled release of flavor and aroma compounds: A critical review. Trends Food Sci. Technol..

[293] Kumar V., Guleria P., Mehta S.K. (2017). Nanosensors for food quality and safety assessment. Environ. Chem. Lett..

[294] George J., Sabapathi S.N. (2015). Cellulose nanocrystals: Synthesis, functional properties, and applications. Nanotechnol. Sci. Appl..

[295] Vong K., Eda S., Kadota Y., Nasibullin I., Wakatake T., Yokoshima S., Shirasu K., Tanaka K. (2019). An artificial metalloenzyme biosensor can detect ethylene gas in fruits and Arabidopsis leaves. Nat. Commun..

[296] Pateiro M., Gómez B., Munekata P., Barba F., Putnik P., Kovačević D., Lorenzo J. (2021). Nanoencapsulation of Promising Bioactive Compounds to Improve Their Absorption, Stability, Functionality and the Appearance of the Final Food Products. Molecules.

[297] Wang L., Hu C., Shao L. (2017). The antimicrobial activity of nanoparticles: Present situation and prospects for the future. Int. J. Nanomed..

[298] Sankar V.R., Reddy Y.D. (2010). Nanocochleate—A new approach in lipid drug delivery. Int. J. Pharm. Pharm. Sci..

[299] Sekhon B. (2014). Nanotechnology in agri-food production: An overview. Nanotechnol. Sci. Appl..

[300] Prakash J., Vignesh K., Anusuya T., Kalaivani T., Ramachandran C., Sudha R.R., Rubab M., Khan I., Elahi F., Oh D.-H. (2019). Ap-plication of Nanoparticles in Food Preservation and Food Processing. J. Food Hyg. Saf..

[301] Ribeiro T., Baleizão C., Farinha J.P.S. (2014). Functional Films from Silica/Polymer Nanoparticles. Materials.

[302] Hsu C.-Y., Wang P.-W., Alalaiwe A., Lin Z.-C., Fang J.-Y. (2019). Use of Lipid Nanocarriers to Improve Oral Delivery of Vitamins. Nutrients.

[303] Pakrashi S., Dalai S., Sabat D., Singh S., Chandrasekaran N., Mukherjee A. (2011). Cytotoxicity of Al_2_O_3_Nanoparticles at Low Exposure Levels to a Freshwater Bacterial Isolate. Chem. Res. Toxicol..

[304] Park E.-J., Lee G.-H., Yoon C., Jeong U., Kim Y., Cho M.-H., Kim D.-W. (2015). Biodistribution and toxicity of spherical aluminum oxide nanoparticles. J. Appl. Toxicol..

[305] Minigalieva I.A., Katsnelson B.A., Privalova L.I., Sutunkova M.P., Gurvich V.B., Shur V.Y., Shishkina E.V., Valamina I.E., Makeyev O.H., Panov V.G. (2018). Combined Subchronic Toxicity of Aluminum (III), Titanium (IV) and Silicon (IV) Oxide Nanoparticles and Its Alleviation with a Complex of Bioprotectors. Int. J. Mol. Sci..

[306] Asharani P.V., Lianwu Y., Gong Z., Valiyaveettil S. (2010). Comparison of the toxicity of silver, gold and platinum nanoparticles in developing zebrafish embryos. Nanotoxicology.

[307] Tao C. (2018). Antimicrobial activity and toxicity of gold nanoparticles: Research progress, challenges and prospects. Lett. Appl. Microbiol..

[308] Boisselier E., Astruc D. (2009). Gold nanoparticles in nanomedicine: Preparations, imaging, diagnostics, therapies and toxicity. Chem. Soc. Rev..

[309] Ahamed M., Siddiqui M., Akhtar M., Ahmad I., Pant A.B., Alhadlaq H. (2010). Genotoxic potential of copper oxide nanoparticles in human lung epithelial cells. Biochem. Biophys. Res. Commun..

[310] Fahmy B., Cormier S.A. (2009). Copper oxide nanoparticles induce oxidative stress and cytotoxicity in airway epithelial cells. Toxicol. Vitro.

[311] Karlsson H.L., Gustafsson J., Cronholm P., Möller L. (2009). Size-dependent toxicity of metal oxide particles—A comparison between nano- and micrometer size. Toxicol. Lett..

[312] Lei R., Wu C., Yang B., Ma H., Shi C., Wang Q., Wang Q., Yuan Y., Liao M. (2008). Integrated metabolomic analysis of the nano-sized copper particle-induced hepatotoxicity and nephrotoxicity in rats: A rapid in vivo screening method for nanotoxicity. Toxicol. Appl. Pharmacol..

[313] de Lima R., Seabra A.B., Durán N. (2012). Silver nanoparticles: A brief review of cytotoxicity and genotoxicity of chemically and biogenically synthesized nanoparticles. J. Appl. Toxicol..

[314] Hadrup N., Sharma A.K., Loeschner K., Jacobsen N.R. (2020). Pulmonary toxicity of silver vapours, nanoparticles and fine dusts: A review. Regul. Toxicol. Pharmacol..

[315] Dos Santos C.A., Seckler M., Ingle A.P., Gupta I., Galdiero S., Galdiero M., Gade A., Rai M. (2014). Silver Nanoparticles: Therapeutical Uses, Toxicity, and Safety Issues. J. Pharm. Sci..

[316] Cao Y., Gong Y., Liao W., Luo Y., Wu C., Wang M., Yang Q. (2018). A review of cardiovascular toxicity of TiO_2_, ZnO and Ag nanoparticles (NPs). BioMetals.

[317] Kumar A., Pandey A.K., Singh S.S., Shanker R., Dhawan A. (2011). Engineered ZnO and TiO_2_ nanoparticles induce oxidative stress and DNA damage leading to reduced viability of Escherichia coli. Free Radic. Biol. Med..

[318] Subramaniam V.D., Prasad S.V., Banerjee A., Gopinath M., Murugesan R., Marotta F., Sun X.-F., Pathak S. (2018). Health hazards of nanoparticles: Understanding the toxicity mechanism of nanosized ZnO in cosmetic products. Drug Chem. Toxicol..

[319] Singh S. (2019). Zinc oxide nanoparticles impacts: Cytotoxicity, genotoxicity, developmental toxicity, and neurotoxicity. Toxicol. Mech. Methods.

[320] Sun Z., Yathindranath V., Worden M., Thliveris J.A., Chu S., Parkinson F.E., Hegmann T., Miller D.W. (2013). Characterization of cellular uptake and toxicity of aminosilane-coated iron oxide nanoparticles with different charges in central nervous system-relevant cell culture models. Int. J. Nanomed..

[321] Sohaebuddin S.K., Thevenot P.T., Baker D., Eaton J.W., Tang L. (2010). Nanomaterial cytotoxicity is composition, size, and cell type dependent. Part. Fibre Toxicol..

[322] Raghunathan V.K., Devey M., Hawkins S., Hails L., Davis S.A., Mann S., Chang I.T., Ingham E., Malhas A., Vaux D.J. (2013). Influence of particle size and reactive oxygen species on cobalt chrome nanoparticle-mediated genotoxicity. Biomaterials.

[323] Morimoto Y., Horie M., Kobayashi N., Shinohara N., Shimada M. (2012). Inhalation Toxicity Assessment of Carbon-Based Nanoparticles. Acc. Chem. Res..

[324] Zhang Y., Petibone D., Xu Y., Mahmood M., Karmakar A., Casciano D., Ali S., Biris A.S. (2014). Toxicity and efficacy of carbon nanotubes and graphene: The utility of carbon-based nanoparticles in nanomedicine. Drug Metab. Rev..

[325] Fröhlich E. (2017). Role of omics techniques in the toxicity testing of nanoparticles. J. Nanobiotechnol..

[326] Hamimed S., Abdeljelil N., Landoulsi A., Chatti A., Aljabali A.A.A., Barhoum A., Barhoum A. (2022). Bacterial Cellulose Nanofibers. Handbook of Nanocelluloses.

[327] Moodley K.G., Arumugam V., Barhoum A., Barhoum A. (2021). Nanocellulose-Based Materials for Wastewater Treatment. Handbook of Nanocelluloses.

